# Co-Delivery of Fluconazole and Riparin B Using Nanostructured Lipid Carriers: A Quality-by-Design Approach for Enhanced Antifungal Activity

**DOI:** 10.3390/pharmaceutics18101229

**Published:** 2026-09-28

**Authors:** Matheus Oliveira do Nascimento, Leandro de Sousa Dias, Allan Kayk Sales Meneses, Sidney Gonçalo de Lima, Carla Veronica Rodarte de Moura, Stanley Juan Chavez Gutierrez, Humberto Medeiros Barreto, Denise Andrade do Nascimento, Gabriel Zazeri, André Luis Menezes Carvalho

**Affiliations:** 1 Postgraduate Program in Pharmaceutical Sciences, Dermopharmacy Laboratory, Pharmaceutical Technology Center, Federal University of Piauí, Teresina 64049-550, PI, Brazil; matheusodn@hotmail.com; 2 Postgraduate Program in Pharmaceutical Sciences, Microbiology Research Laboratory, Department of Parasitology and Microbiology, Federal University of Piauí, Teresina 64049-550, PI, Brazil; ldias@ufpi.edu.br (L.d.S.D.); hmbarreto@ufpi.edu.br (H.M.B.); 3 Postgraduate Program in Chemistry, Organic Geochemistry Laboratory, Department of Chemistry, Federal University of Piauí, Teresina 64049-550, PI, Brazil; allankayk@ufpi.edu.br (A.K.S.M.); sidney@ufpi.edu.br (S.G.d.L.); 4 Postgraduate Program in Chemistry, Biodiesel and Materials Laboratory, Department of Chemistry, Federal University of Piauí, Teresina 64049-550, PI, Brazil; carla@ufpi.edu.br; 5 Undergraduate Course in Pharmacy, Department of Pharmacy, Federal University of Piauí, Teresina 64049-550, PI, Brazil; stanleychavez@ufpi.edu.br; 6 Department of Physics, Federal University of Roraima (UFRR), Boa Vista 69310-000, RR, Brazil; denise.nascimento@ufrr.br

**Keywords:** fluconazole, riparin B, nanostructured lipid carriers, quality by design, molecular docking

## Abstract

**Background/Objectives**: The increasing incidence of invasive fungal infections and the emergence of antifungal resistance have limited the effectiveness of conventional therapies against *Candida* spp. This study aimed to develop and optimize nanostructured lipid carriers (NLCs) containing fluconazole (FLC) and Riparin B (RIP) using a systematic Quality-by-Design (QbD) approach. **Methods**: The optimized NLCs were characterized by particle size, polydispersity index (PDI), zeta potential, DSC, and FTIR analyses. Molecular docking was used as a complementary exploratory approach to investigate possible interactions of RIP with representative components of the lipid matrix and with fungal resistance-associated proteins. Release profiles and antifungal activity against *Candida* spp. were also evaluated. **Results**: The optimized NLCs exhibited a mean particle size of 144.9 ± 2.25 nm, PDI of 0.153 ± 0.03, and zeta potential of −27.8 ± 1.81 mV. DSC and FTIR analyses supported the structural organization of the lipid matrix and the formation of an imperfect crystalline network, providing structural defects favorable for the incorporation of the active compounds. RIP exhibited more favorable predicted interactions than FLC within the specific lipid configuration evaluated, although these docking scores should not be interpreted as direct thermodynamic measurements of drug–lipid affinity or as quantitative predictors of encapsulation efficiency. Docking analyses with MdR1, CdR1, and CdR2 identified possible interactions of RIP with these resistance-associated proteins, providing a structural hypothesis that may contribute to understanding the potentiation of FLC activity. However, these computational results do not establish direct efflux-pump inhibition. Release profiles followed distinct kinetic regimes, consistent with matrix relaxation for RIP and Fickian diffusion for FLC. Although RIP alone did not exhibit relevant antifungal activity, its association with FLC potentiated FLC activity against *Candida* spp., while incorporation of the combination into NLCs produced a further enhancement, significantly reducing the minimum inhibitory concentration. **Conclusions**: These findings indicate that the optimized NLC platform can enhance the antifungal performance of the FLC–RIP combination and provide a formulation framework for further investigation of this co-delivery strategy.

## 1. Introduction

The epidemiology of *Candida* infections has changed in recent years, posing a significant challenge to public health. These infections are associated with high mortality rates and substantial healthcare costs, placing a significant burden on healthcare systems and hospitalized patients [1,2,3]. Given the limited number of available antifungal drug classes, the emergence of resistance to single and multiple antifungal agents further complicates treatment [4,5]. Fluconazole (FLC) is a widely used azole antifungal drug for the treatment of *Candida* infections. However, prolonged and widespread use of FLC has contributed to the development of resistance [6,7].

An important mechanism of resistance to FLC is reduced intracellular drug accumulation, mediated by transmembrane proteins known as efflux pumps [8,9]. Efflux pump inhibitors (EIs) are emerging as promising adjuvants that block the efflux of antifungals and chemosensitize drug-resistant strains. The discovery of new inhibitors may therefore lead to the development of combination therapies that enhance the efficacy of azoles [10,11,12].

N-benzoyltyramines, known as riparins, are natural alkaloids isolated from Aniba riparia (Nees) Mez (Lauraceae). In addition to the natural forms of these molecules, chemical synthesis has become the predominant method for obtaining analogues, such as riparin-B (N-[2-(3,4-dimethoxyphenyl)ethyl] benzamide, RIP) [13,14]. RIP exhibits several biological activities and has been particularly recognized as an important efflux pump inhibitor (EI). Previous studies have shown that RIP can enhance the efficacy of norfloxacin and ciprofloxacin against strains of *Staphylococcus aureus* and *Acinetobacter baumannii*, possibly through inhibition of efflux mediated by the NorA [15] and AdeABC proteins [16], respectively.

Nanostructured lipid carriers (NLCs) are among the most promising lipid-based nanosystems, particularly for the treatment of fungal skin infections [17,18]. These systems consist of a low-crystallinity solid lipid matrix, formed by combining solid and liquid lipids, dispersed in an aqueous phase stabilized by one or more surfactants [19,20]. Different lipid sources have been explored for NLC composition. Among these, natural lipids have gained prominence because they combine intrinsic biological properties with advantages related to safety, abundance, low cost, and structural diversity, often offering benefits over synthetic lipids [21,22,23,24,25].

The Quality-by-Design (QbD) approach has been widely applied to optimize lipid-based nanosystem formulations and is increasingly required by regulatory authorities [26,27]. Design of Experiments (DoE), one of the tools used in QbD, involves a systematic variation in input factors to determine their effects on the measured responses, allowing the identification of the most influential input factors, the determination of the factor combination that optimizes the responses, and the understanding of interactions between different input factors [28,29].

Thus, the present study employed a systematic Quality-by-Design (QbD) approach combined with Response Surface Methodology (RSM) to develop and optimize natural lipid-based nanostructured lipid carriers (NLCs) containing fluconazole (FLC) and Riparin B (RIP-B), with the aim of improving antifungal performance against *Candida* spp. Previous research has investigated lipid-based nanoparticles containing FLC and RIP-B, including their simultaneous spectrophotometric determination, encapsulation, and release behavior [30]. However, the antifungal activity of this combination against *Candida* spp. was not evaluated in that study. In a separate investigation, da Silva et al. [31] evaluated the in vitro inhibitory activity of riparins I–IV against *Candida* spp. and explored, through molecular modeling, their potential interactions with multidrug-resistance-associated proteins. Although this study provided relevant insights into the antifungal potential of riparins and their predicted molecular interactions, it did not investigate RIP-B in combination with FLC within a nanostructured lipid carrier system. Given the structural differences among riparins, the findings obtained for riparins I–IV cannot be directly extrapolated to RIP-B. Accordingly, the potential of RIP-B to enhance the antifungal activity of FLC against *Candida* spp., particularly when incorporated into a lipid-based nanocarrier, remains insufficiently characterized. Building on these previous findings, the present study investigated RIP-B as a potential adjuvant to FLC and integrated its antifungal evaluation with the systematic QbD/RSM-driven development of an NLC formulation based on natural lipids. Specifically, we assessed the effects of formulation and process variables on critical quality attributes, characterized the optimized formulation, evaluated its drug-release behavior, and compared the antifungal activity of free and NLC-encapsulated FLC–RIP-B against *Candida* spp. Molecular modeling was also employed as a complementary exploratory approach to investigate potential interactions with resistance-associated proteins, without assuming direct efflux inhibition or a specific resistance-modulating mechanism. Collectively, this study provides an integrated assessment of RIP-B as a potential antifungal adjuvant to FLC and its incorporation into a systematically optimized NLC platform, combining formulation development, physicochemical characterization, drug-release evaluation, antifungal testing, and exploratory molecular modeling.

## 2. Materials and Methods

### 2.1. Materials

All reagents used were of analytical grade or higher. FLC was purchased from Sigma-Aldrich (St. Louis, MO, USA). RIP was kindly provided by Prof. Dr. Stanley Juan Chavez Gutierrez of the Federal University of Piauí (Teresina, PI, Brazil) and synthesized according to previously reported methods [32,33]. Bacuri butter (*Platonia insignis* Mart.) was purchased from Amazon Oil Industry (Ananindeua, PA, Brazil). Oleic acid was purchased from Synth (Diadema, SP, Brazil). Pluronic^®^ F127 was obtained from ChemSpecs (São Paulo, SP, Brazil).

### 2.2. Preparation of NLCs

NLCs were prepared by hot emulsification followed by ultrasonication [24]. The oil phase, composed of solid lipid and liquid lipid, was heated to 10 °C above the melting point of the solid lipid (50 ± 5 °C). The aqueous phase, containing the surfactant, was heated to the same temperature and then added to the oil phase. The resulting pre-emulsion was then subjected to high-intensity ultrasonication using an ultrasonic processor (Eco-sonics QR500, 20 kHz, Ultronique, Indaiatuba, Brazil). Finally, the emulsion was cooled to room temperature to solidify the lipid and form the nanoparticles. To prepare the formulations with fluconazole and riparin-B (NLC-FR), the drugs were added to the oil phase after heating.

### 2.3. Establishing QbD Parameters

The following QbD steps were performed: (I) definition of the Quality Target Product Profile (QTPP); (II) identification of potential Critical Quality Attributes (CQAs); (III) defining potential Critical Process Parameters (CPPs) and Critical Material Attributes (CMAs); (IV) initial risk assessment and factor screening studies; (V) optimization and identification of the optimal conditions for drug encapsulation [34]. The QbD parameters, especially the definition of the QTPP and CQAs, were established based on knowledge obtained from the literature, previous experience, and experimental evaluations of the formulation.

The initial risk assessment was conducted by drawing an Ishikawa diagram based on the identified CMAs and CPPs, and using the Failure Mode and Effects Analysis (FMEA) method. In FMEA, failure modes were prioritized based on three attributes: severity, occurrence, and detectability. The Risk Priority Number (RPN) was calculated to classify the relative risk of each variable, according to the following Equation (1) [35,36]:

RPN = S × O × D(1)

where S represents severity, a measure of how seriously a failure mode affects the product, O represents the probability of an event occurring, and D represents detectability, or the ease with which a failure mode can be detected [37].

#### Design of Experiments (DoE)

Based on the evaluation of effects during the screening stage of CMAs and CPPs, the factors influencing CQAs were identified (Table 1). The most critical and relevant factors were selected for optimization, whereas the remaining factors were maintained at predefined levels according to the central composite design (CCD). In this experimental optimization plan, the relationships between CMAs and CPPs and their effects on responses (CQAs) were evaluated to determine the most appropriate levels for each factor. Each experimental condition was evaluated in triplicate using random sampling. Models describing the influence of the factors were obtained using Design-Expert^®^ 12.0.1.0 software (test version Stat-Ease Inc., Minneapolis, MN, USA) and verified using ANOVA [38].

After obtaining the appropriate regression models for each CQA, the combination of factors with the highest desirability value was selected as the ideal condition. Next, three NLC formulations were prepared to validate the model equations by comparing predicted and observed response values [39].

### 2.4. Characterization of NLCs

#### Determination of Particle Size, Polydispersity Index, and Zeta Potential

The mean hydrodynamic diameter and polydispersity index (PDI) of the NLCs were measured at 25 °C by dynamic light scattering (DLS). The measurements were performed using a Zetasizer Nano ZS90 (Malvern Instruments Ltd., Malvern, UK). The zeta potential was estimated by measuring the electrophoretic mobility of the particles using the same equipment. Before analysis, all samples were diluted 1:500 (*v*/*v*) with ultrapure water. The data represent the average of three measurements [40].

### 2.5. Incorporation of FLC and RIP in the Optimized Formulation

#### 2.5.1. Compatibility Study Between Drugs

The compatibility study was conducted to analyze the molecular structures and identify possible interactions between FLC and RIP. For this purpose, the drugs were evaluated individually and in a 1:1 (*w*/*w*) ratio by attenuated total reflectance Fourier transform infrared spectroscopy (ATR-FTIR) [41], using an Agilent Cary 630 FTIR (Agilent Technologies Inc., Santa Clara, CA, USA) with an ATR accessory (diamond crystal, single reflection, top plate, and pressure arm) using Agilent MicroLab software 5.8. The spectra were recorded in transmittance mode in the mid-infrared region (4000–650 cm^−1^). The number of scans was 64, with a resolution of 2 cm^−1^.

#### 2.5.2. Computational Modeling

To understand the possible interactions between the components of the lipid matrix and the drugs, molecular docking simulations were performed using AutoDock 4.2 software [42]. Before the simulations, the compound structures were optimized using Chem3D 16.0 after being drawn in ChemDraw Ultra 16.0 or obtained from the NCBI (National Center for Biotechnology Information). The energies of the structures were minimized and then converted to .pdb format.

##### Construction of the Lipid Matrix

To simulate the three-dimensional lipid matrix, the number of molecules of the lipid mixture components was adjusted to match the proportions of the main triacylglycerols present in bacuri butter [43]. Because the actual number of molecules would be prohibitively large, the total number of molecules was determined by calculating the mass of each triacylglycerol, dividing it by the corresponding molecular weight, and then multiplying by Avogadro’s number to obtain the total number of molecules for the simulated system [44]. Thus, the system was constructed using the following compounds: tripalmitin (27 molecules), 1,3-dipalmitoyl-2-oleoylglycerol (32 molecules), 3-Palmitoyl-1,2-dioleyn (12 molecules), and oleic acid (42 molecules). The model was created using Packmol software 21.2.3 [45] and visualized using VMD 1.9.3 (Visual Molecular Dynamics) [46].

##### Molecular Docking

Molecular docking was performed to investigate the interactions of the drugs with the lipid matrix and to obtain docking scores. FLC and RIP were treated as ligands, with the lipid mixture functioning as the receptor. Up to 100 docking conformations per ligand were generated. Kollman charges and hydrogens necessary for the calculation were added. The rotatable bonds of the ligands were defined automatically. The conformations were ranked according to the docking scoring function implemented, using the Lamarckian Genetic Algorithm. After identifying potential binding sites, the complex conformations were optimized using the steepest-descent algorithm until convergence, with a maximum of 20 interactions [47]. The grid box was set to contain 70, 70, and 70 points in the x, y, and z dimensions, respectively, with a grid spacing of 1.000 Å and a grid center at coordinates 34.414, 34.027, and 36.652 Å. The conformations were analyzed using Discovery Studio imaging software (Accelrys, Inc., San Diego, CA, USA) to investigate the interactions between the ligands and the lipid matrix. The numerical values obtained from the docking calculations are reported as docking scores and were used for comparative analysis of the predicted ligand–lipid matrix interactions.

##### Consensus Docking

Molecular docking simulations were performed to investigate the possible mechanism of action of RIP against *Candida* spp. by analyzing its predicted interactions with proteins associated with antifungal resistance. The binding mode and docking scores of RIP against the main efflux transporters, Mdr1 (UniProtKB–Q5ABU7), Cdr1 (UniProtKB-Q5ANA3), and Cdr2 (UniProtKB—P78595), were evaluated. FLC was used as a control in the simulations [48].

Additionally, a consensus analysis was performed using multiple scoring functions to reduce false positives. In the consensus calculation, the compounds’ predicted interactions were assessed using the Vina Score, MolDock Score, Rerank Score, and PLANTS Score. These scoring functions were obtained using Molegro Virtual Docker (MVD) v. 6.0.1. The following parameters were used: 10 runs, a maximum of 1500 interactions, and a population of 50 individuals. For each flexible residue, 2000 minimization steps were performed, and the same number of steps was applied to global minimization by simulation. The grid (GRID) was defined with a spacing of 0.3 Å, and the search sphere was set with a radius of 15 Å [31].

For each scoring function analyzed, the value of (*p*) was calculated, corresponding to the ratio between the score of each compound tested and the score of the compound that obtained the lowest energy. The second consensus analysis consisted of calculating the overall average considering all biomarkers studied. Thus, the consensus values for each compound were determined as an average of the probabilities obtained for each biomarker. After obtaining the *p* values for the compounds in each scoring function, the total consensus value (*p*) was calculated according to Equation (2):

Penzyme = (PVinaScore+ PMolDockScore+ PRerankScore+ PPLANTS Score)/*n* (2)

#### 2.5.3. Solubility Study

Given the hydrophilic nature of FLC, its solubility in the optimized lipid composition was evaluated to determine the maximum concentration suitable for incorporation into the NLCs, following the method described by Kasongo et al. [49], slightly modified. The experiment consisted of adding increasing amounts of FLC (0.1, 0.2, and 0.3% *w*/*w*) to the optimized lipid composition, heating the mixture to 50 °C to melt the lipid, and agitating at 300 rpm for 30 min. The solubility of FLC in the melted lipid was evaluated visually, verifying the formation of a clear solution and the presence of residual crystals by PLM [50]. The RIP concentration was determined after preparation of NLC-FR, based on the maximum amount of FLC observed upon visualizing precipitates in the dispersion.

#### 2.5.4. Drug Recovery, Encapsulation Efficiency, and Encapsulation Capacity

To obtain the total FLC and RIP (free drug plus encapsulated drug, TC) content in the NLCs, an aliquot of the dispersion was diluted in ethanol, sonicated (Unique, Idaiatuba, Brazil) for 10 min, and centrifuged (Cientec CT-6000 centrifuge) at 3000 rpm for 15 min. Then, the drugs in the supernatant were quantified using a UV–Vis spectrophotometer. Drug recovery (DR%) was calculated by comparing the measured TC with the amount of drug initially used to prepare the nanoparticles [51].

The encapsulation efficiency (EE%) and encapsulation capacity (DL%) were determined by an indirect method, quantifying the free, unencapsulated drug. For this, an aliquot of the NLC-FR dispersions was diluted 1:1 (*v*/*v*) in water and then centrifuged using an Amicon Ultra-15 MWCO 10,000 filter (Merck Millipore, Darmstadt, Germany) for 15 min at 5000 rpm in an NT 820 centrifuge (Novatecnica, Piracicaba, Brazil). The drug in the filtrate (FF) was quantified with a UV–Vis spectrophotometer. EE% and DL% were calculated according to the following equations (Equation 3 and Equation 4, respectively): [52]

EE% = [(TC − FF)/TC] × 100(3)

DL% = [(TC − FF)/TL] × 100(4)

where TL is the total amount of lipids used in the formulation.

All quantifications were performed using a UV–Vis spectrophotometer (UV-1800, Shimadzu, Kyoto, Japan) with a previously validated method [30].

#### 2.5.5. FTIR

The interactions between the drugs and lipids were also evaluated using ATR-FTIR spectroscopy [53]. Before analysis, the aqueous dispersions of NLCs and NLC-FR were frozen overnight and then lyophilized (Liotop L101, Liobras, São Carlos, Brazil) for 48 h using mannitol (10% *w*/*w*) as a cryoprotectant [54]. Subsequently, the infrared spectra of the samples (bacuri butter, oleic acid, Pluronic^®^ F127, NLCs, and NLC-FR) were obtained using the method described in Section 2.5.1.

#### 2.5.6. DSC

The thermal properties of the samples were determined from the curves obtained by differential scanning calorimetry (DSC) using DSC-60 equipment (Shimadzu). Samples (3 mg) were placed in platinum crucibles and heated from 25 to 300 °C at a heating rate of 10 °C/min under a nitrogen atmosphere with a flow rate of 50 mL/min. The crystallinity index (CI) was calculated from the melting enthalpy using the following Equation (5):

CI (%) = (ΔHNLC/ΔHsolidlipid) × 100(5)

where ΔHNLC and ΔHsolidlipid correspond to the enthalpies of fusion of the nanoparticles and the solid lipid used in the formulation, respectively [21].

### 2.6. Physical Stability During Storage Time

To evaluate the long-term stability of NLC-FR, samples were stored in sealed amber bottles and kept in a refrigerator at 5 ± 2 °C for 90 days. At predetermined intervals (0, 30, 60, and 90 days), possible changes in mean hydrodynamic diameter, PDI, zeta potential, and EE% were observed.

### 2.7. In Vitro Release of FLC and RIP

The in vitro release study of FLC and RIP from the NLC-FR formulation, as well as from a solution of FLC and RIP (FLC-RIP), was performed using Franz-type diffusion cells (diffusion area of 1.72 cm^2^) connected to a thermostatic water bath [55]. A 12–14 kDa MWCO dialysis membrane (Servapor, Heidelberg, Germany) was used between the donor and recipient compartments. The donor compartment was filled with 1 mL of the samples (containing 2000 μg of FLC and 1000 μg of RIP for NLC-FR, and 1000 μg of FLC and 500 μg of RIP for FLC-RIP, the latter being solubilized in the receptor medium), while the recipient compartment contained 15 mL of a mixture of phosphate buffer (pH 7.4) and ethanol (80:20) [56]. The experiment was performed under stirring at 500 rpm at 32 ± 0.5 °C. Aliquots (3 mL) of the receiving medium were collected and replaced with fresh, preheated medium at 0.5, 1, 2, 4, 6, 8, and 10 h after sample application. The concentrations of FLC and RIP at all times were determined using a UV–Vis spectrophotometer with a previously validated method [30]. The in vitro release profiles were fitted using the DDSolver add-in for Microsoft Excel^®^ for Microsoft Excel^®^ to different mathematical models. The quality of the adjustment for each model was evaluated using the adjusted coefficient of determination (R2adj), Akaike information criterion (AIC), and model selection criterion (MSC) [57].

### 2.8. Antifungal Activity

The reference strains were obtained from the microbiological collection of the Microbiology Research Laboratory at the Federal University of Piauí, where they are stored in solid Sabouraud Dextrose/Chloramphenicol Neogen agar at 8 °C. The reference strains *C. albicans* ATCC 10231, *C. tropicalis* ATCC 28707, *C. krusei* ATCC 34135, *C. glabrata* ATCC 2001, and *C. parapsilosis* ATCC 22019 were used to evaluate the antifungal activity of the tested substances. All microorganisms were seeded and incubated in an oven at 37 °C in 3% Brain Heart Infusion (BHI) medium 24 h before the experiments. Each microbial culture was suspended in saline solution (0.85%), and the fungal inoculum was initially standardized to approximately 1.5 × 10^6^ CFU/mL by adjusting the optical density adjusted to 0.5 on the MacFarland scale using a Kasvi K37-VIS spectrophotometer (São José dos Pinhais, Paraná, Brazil).

Antifungal activity was evaluated using the broth microdilution method as described by Leal et al. [58]. Each microbial culture was diluted in saline solution (NaCl 0.85%); then the fungal inoculum was initially standardized to approximately 1.5 × 10^6^ CFU/mL using a Kasvi K37-VIS spectrophotometer (São José dos Pinhais, Paraná, Brazil). After initial standardization, 100 µL of the fungal suspension was diluted with 900 µL of BHI broth, resulting in a 1:10 dilution and an estimated concentration of approximately 1.5 × 10^5^ CFU/mL. Subsequently, 100 µL of the diluted inoculum was added to the wells of a 96-well plate, followed by 100 µL of the respective test solution (FLC, RIP, FLC-RIP, NLCs, NLC-FR) at concentrations ranging from 1024 to 0.5 μg/mL using two-fold serial dilutions. After homogenization, 100 µL of the resulting mixture was removed, resulting in a final volume of 100 µL per well and an estimated final inoculum concentration of approximately 7.5 × 10^4^ CFU/mL. A growth control containing the standardized fungal inoculum without the test substance was included in each assay. The plates were incubated at 37 °C for 24 h. The minimum inhibitory concentration (MIC) was defined as the lowest concentration at which no visible microbial growth was observed. All experiments were performed in triplicate, and the results are expressed as geometric means.

The minimum fungicidal concentration (MFC) was determined according to Leal et al. [58], with adaptations. Aliquots of 5 µL of the dilutions tested by microdilution were inoculated into Sabouraud Dextrose/Chloramphenicol Agar medium, followed by incubation at 37 °C for 24 h. After this period, the plates were examined to determine whether microbial growth was observed. The MFC was defined as the lowest concentration of the test substance at which no microbial growth was observed. All experiments were performed in triplicate, and the test results are expressed as the geometric mean.

### 2.9. Statistical Analyses

All quantitative results are expressed as mean ± standard deviation (SD). Statistical analysis of the data was performed using GraphPad Prism 8.0 software. One-way analysis of variance (ANOVA) was used to assess differences among datasets, followed by Tukey’s or Bonferroni’s multiple-comparison test, as appropriate. The level of statistical significance was set at *p* < 0.05.

## 3. Results and Discussion

### 3.1. Definition of QTPP and CQAs

The QTPP (Table S1, Supplementary Material) defined the target attributes required to ensure NLCs’ quality, efficacy, and stability, focusing on critical parameters such as particle size, PDI, drug content, and release profiles.

The CQAs were derived from the QTPP and defined based on information from the literature on lipid nanoparticles, regardless of the type of lipid used in the composition. This distinction is relevant because synthetic lipids composed primarily of specific isolated substances are more commonly used. The solid lipids investigated in this article are composed of a mixture of fatty acids with different chemical structures and melting points. Table S2 (Supplementary Material) illustrates the CQAs that affect the quality of NLCs, along with the justification for each of them.

### 3.2. Initial Quality Risk Assessment Study

The Ishikawa diagram illustrates the factors that may affect formulation quality and, consequently, its final performance. The factors were divided into five categories: people, equipment, process variables, materials, methods, and environment, identified through prior knowledge of the formulation and production process (Supplementary Material Figure S1).

After mapping the factors that may affect CQAs during the formulation stage, a risk estimation matrix (REM) was constructed to assign low (green), medium (yellow), and high (red) risk levels to each NLC material and process variable, as well as to the QTPP variables. The REM suggested that CMAs pose a higher risk than CPPs (Supplementary Material Figure S2). The factors were then prioritized for risk management using FMEA, which was used to identify and assess potential failure modes, prioritized by CMAs and CPPs that affect the formulation’s CQAs (Supplementary Material Table S3).

### 3.3. Formulation Optimization

After identifying the significant factors during the screening phase, response surface methodology was applied to generate experimental data for optimization using CCD. Some variables were fixed based on the results obtained in the screening stage. A summary of the variables used and the measured responses used in the CCD is presented in Table 2.

The quadratic model was considered the most appropriate for analyzing the responses (Y). The significance of the linear, quadratic, and interaction terms was calculated using ANOVA (Supplementary Material Table S4). The regression equations (Supplementary Material Table S5) were determined by evaluating *p*-values, and the calculated coefficients of determination (R2adj and R2pred) were used to assess the adjusted multiple regression model [59].

#### 3.3.1. Effect of Factors on Particle Size (Y1)

The formulations showed average sizes in the nanometer range (125.4 ± 2.50 to 213.6 ± 2.37 nm), with less variation in particle size than the NLCs previously prepared during the screening stage, due to the fixed factors. All factors (A, B, and C) significantly influenced the response (Y1) (*p* < 0.05). In addition, the effect graphs (Supplementary Material Figure S3) indicate that an increase in any individual factor resulted in a reduction in particle size.

Table S5 (Supplementary Material) reveals that surfactant concentration was the factor most influencing particle size, as indicated by the highest coefficient in the regression equation. This is due to the surfactant’s ability to adsorb onto the particle surface and reduce the interfacial tension between the lipid and aqueous phases [60]. However, this effect is nonlinear, as evidenced by the significant and antagonistic quadratic effect (B2). The same applies to sonication time (C), with a significant and antagonistic quadratic effect (C2).

Particle size was inversely related to the amount of liquid lipid, indicating a linear reduction in particle size as the amount of liquid lipid increases. The incorporation of more liquid lipid into the system can reduce viscosity and surface tension, thereby facilitating a decrease in particle size [61].

#### 3.3.2. Effect of Factors on the Polydispersity Index (Y2)

The formulations exhibited PDI values ranging from 0.1 ± 0.02 to 0.235 ± 0.03, representing a narrow size distribution and good homogeneity (PDI < 0.3). The surfactant concentration (B) and sonication time (C) showed a significant effect on PDI (Y2) (Supplementary Material Table S4). A higher concentration of Pluronic F127 yields a more polydisperse particle size, whereas a longer sonication time resulted in a more homogeneous, albeit nonlinear, size distribution (C2: *p* < 0.05). An increase in surfactant concentration increases the PDI due to particle aggregation caused by surface-bound surfactants [62].

Although changes in liquid lipid concentrations (linear term) do not significantly affect the PDI, its quadratic term (A2) exhibits a significant synergistic effect on the response (Y2). This indicates that increasing the amount of oleic acid may initially decrease PDI values up to a specific concentration. However, as the concentration approaches the maximum level (+1), the PDI increases again, returning to the initial values. This effect may indicate that the reduction in viscosity caused by the liquid lipid is an essential factor in reducing particle size and PDI. However, an increase in the amount of liquid lipid may increase hydrophobicity, leading to slight particle aggregation [63].

The response (Y2) is also significantly affected (*p* < 0.05) by the interaction between the liquid lipid concentration (A) and the sonication time (C). This indicates that the reduction in PDI as a function of sonication time (factor C) depends on the level of liquid lipid concentration (factor A). At lower oleic acid concentrations, increasing the sonication time yields greater uniformity in particle size. On the other hand, at higher oleic acid concentrations, increasing the sonication time tends to slightly raise the PDI values, as shown in Figure 1.

#### 3.3.3. Effect of Factors on Zeta Potential (Y3)

The Y3 response values ranged from −24.7 to −24.3 ± 1.8 mV, as shown in Table 2. Although the regression coefficient of the equation (Supplementary Material Table S5) does not indicate good predictive power, the quadratic terms for liquid lipid concentration (A^2^) and sonication time (C^2^) were significant. Both showed a positive relationship with the response, as illustrated in Figure S3 (Supplementary Material).

This is likely because a higher oleic acid content leads to the formation of spherical nanoparticles, which facilitates the surfactant’s adsorption to the surface. This process replaces the endogenous surface-active compounds of NLCs, which are responsible for the negative zeta potential, resulting in less negative values. In contrast, nanoparticles with lower oleic acid content tend to have irregular, quasi-spherical, cylinder-like shapes, which results in lower surfactant adsorption and, consequently, higher liquid lipid content on the particle surface [64]. The same effect is observed with respect to sonication time.

### 3.4. Optimized Formulation and Validation

An optimized NLC formulation was obtained based on the CCD results. Based on the regression models, the optimal formulation conditions were selected using desirability functions to maximize the desired responses. The optimization criteria were to minimize particle size and PDI. The zeta potential was adjusted to remain within the observed experimental limits, since the surfactant used is nonionic, thereby preventing particle aggregation through steric rather than electrostatic effects. Thus, the final parameters of the optimized NLCs were defined as 24.9% liquid lipid (relative to the total amount of lipids), 4.47% surfactant (relative to the aqueous phase), and a sonication time of 9.39 min.

The comparison between the predicted values and the values obtained for all responses reveals a prediction error percentage of less than 5%, indicating the suitability of the selected models for the optimization of the process (CPPs) and materials (CMAs) for the production of natural lipid-based NLCs (Supplementary Material Table S6).

### 3.5. Incorporation of FLC and RIP in the Optimized Formulation

#### 3.5.1. Study of Compatibility Between Drugs

FTIR was used to evaluate, for the first time, the compatibility between FLC and RIP, to ensure that the combination of drugs did not result in physicochemical interactions that could modify the stability of the formulation, molecular identity, or pharmacological effect [39]. The spectra of FLC, RIP, and the physical mixture (FLC + RIP) are shown in Figure 2.

FLC was identified based on the characteristic vibrational bands of its molecular structure, highlighting a broad band in the region between 3400 cm^−1^ and 2900 cm^−1^, attributed to the stretching of O—H bonds due to intramolecular and intermolecular hydrogen bonds, as well as the stretching of C—H bonds [65]. The bands at 1620 cm^−1^ and 1591 cm^−1^ correspond to the C=C stretching of the aromatic ring, while the band at 965 cm^−1^ is related to the out-of-plane bending (deformation) of the aromatic ring. The bands at 1510 cm^−1^, 1420 cm^−1^, and 1081 cm^−1^ are associated with the stretching of triazole rings. The stretching vibrations of C—F bonds can be observed in the bands at 1276 cm^−1^ and 1138 cm^−1^ [66,67,68]. RIP was primarily identified by the characteristic stretching vibrations of the N–H, C=O, and C–N bonds, with bands at 3335 cm^−1^, 1630 cm^−1^, and 1025 cm^−1^, respectively, characterizing the presence of amide groups. The bands at 1295 cm^−1^ and 1233 cm^−1^ are associated with C—O stretching, typical of ether vibrations in substituted aromatic rings, such as the methoxy groups present in the RIP structure. In addition, angular deformation out of the plane of the C—H bond of the monosubstituted rings can be observed at 764 cm^−1^. The band at 1517 cm^−1^ refers to the C=C stretching of the aromatic ring, while the band at 2935 cm^−1^ corresponds to the stretching of the aliphatic C—H bonds, due to the two methoxy groups located in the phenyl ring [69,70].

For the physical mixture (FLC + RIP), the spectra showed overlapping bands, allowing identification of the characteristic functional groups of each drug. The presence of FLC in the mix is evidenced by five main markers: O—H stretching at 3110 cm^−1^, characteristics of triazole rings in the bands at 1420 cm^−1^ and 1081 cm^−1^, C—F stretching in the band at 1138 cm^−1^, and angular deformation outside the plane of the aromatic ring at 965 cm^−1^. The presence of RIP is identified by six structures: stretching of the N—H, C=O, and C—N bonds of the amide group in the bands at 3335 cm^−1^, 1630 cm^−1^ and 1025 cm^−1^, C—O stretching in the band at 1233 cm^−1^, which identifies the presence of methoxy groups, angular deformation out of the plane of the C—H bond at 764 cm^−1^ and the band at 1517 cm^−1^ of C=C stretching.

Thus, the results demonstrate no changes in the chemical structures of the drugs in the physical mixture, suggesting chemical compatibility. This finding is important because it indicates that FLC and RIP can be combined in a single formulation without compromising stability and therapeutic efficacy.

#### 3.5.2. Molecular Docking of Drugs with the Lipid Matrix

In silico modeling of nanocarriers has now become a powerful virtual screening tool for optimizing new drug delivery systems [71].

Molecular docking was performed as an exploratory computational approach to investigate possible molecular interactions of FLC and RIP with representative components of the optimized lipid matrix (Figure 3). The lipid model was constructed using Packmol and comprised the triglyceride components and oleic acid described in Section Construction of the Lipid Matrix. Because the resulting structure represents an initial molecular configuration rather than an equilibrated and dynamically sampled lipid phase, the docking results should be interpreted qualitatively and should not be considered direct measurements of thermodynamic drug–lipid affinity.

The docking calculations resulted in docking scores of −4.61 kcal/mol for FLC and −6.41 kcal/mol for RIP. The more negative predicted docking score for RIP indicates a more favorable docking score within the specific molecular configuration evaluated in the model. However, these values should not be interpreted as quantitative thermodynamic free energies or as direct predictors of encapsulation efficiency, drug loading, or drug-release behavior. Rather, the results provide qualitative evidence that RIP can establish favorable molecular contacts with the modeled lipid components.

The predicted interactions also differed between the two compounds. FLC predominantly exhibited hydrophobic interactions, including π-alkyl and π-sigma contacts, mainly involving PPP and POP. In contrast, RIP established a combination of hydrogen-bonding and hydrophobic interactions, including alkyl and π-alkyl contacts, with several lipid components, including PPP, POP, and POO. The predicted interaction distances ranged from approximately 2.64 to 5.31 Å. No relevant interaction between RIP and oleic acid was identified in the analyzed docking poses.

These results suggest that RIP may establish a greater diversity of molecular contacts with the modeled lipid components than FLC. This observation may be consistent with differences observed experimentally in the encapsulation behavior of the two compounds. Nevertheless, the docking analysis does not establish a causal or quantitative relationship between the calculated docking scores and encapsulation efficiency, drug loading, or drug-release kinetics. The experimentally observed differences in encapsulation and release should therefore be interpreted primarily in the context of the physicochemical characteristics of the drugs and the experimentally characterized properties of the NLC formulation.

It is also important to acknowledge that a lipid matrix is intrinsically dynamic and heterogeneous and does not necessarily contain rigid binding pockets analogous to those observed in protein structures. Consequently, the predicted interactions may depend on the initial molecular configuration generated by Packmol and on the conformational sampling accessible to the lipid components. Molecular dynamics simulations using equilibrated lipid systems, independent replicates, and appropriate analyses of drug distribution, partitioning, or free-energy profiles would be required to provide a more rigorous assessment of drug–lipid interactions. Such analyses are beyond the scope of the present study and should be considered in future investigations.

Therefore, the docking results are presented here as complementary and exploratory structural evidence supporting the possibility of favorable interactions between RIP and the lipid matrix, rather than as direct thermodynamic evidence of drug encapsulation or drug–lipid affinity.

#### 3.5.3. Solubility Study

Considering the results of molecular docking and the importance of high drug solubility in the melted lipid to ensure satisfactory encapsulation capacity and greater encapsulation efficiency [50,72], the solubility of FLC in the optimized lipid composition was determined visually and microscopically (under polarized light). In Figure S4 (Supplementary Material), FLC was not completely solubilized in the lipid phase, even at the lowest concentration tested (0.1%). As the drug concentration increases, the presence of crystals becomes more evident (red arrows). The incomplete solubilization of FLC in the optimized lipid phase provides experimental support for its limited incorporation into the lipid matrix and is consistent with the lower EE% observed for FLC [73].

Based on the results, concentrations of 0.1% (*w*/*w*) and 0.2% (*w*/*w*) FLC were selected for incorporation into the NLCs due to the lower crystal content. For comparison purposes and to adjust some physicochemical characteristics of the resulting NLCs, NLC-FR were prepared together with RIP at 0.1% (*w*/*w*) (maximum amount that did not result in precipitation).

A higher EE% for RIP than for FLC was expected, since FLC is highly hydrophilic (log *p* = 0.5) due to the presence of a hydroxyl group and two triazole rings, which makes it less lipophilic than other azole derivatives (Table 3) [74]. In addition, the NLC preparation process favors partitioning of the hydrophilic drug into the aqueous phase during homogenization, resulting in low EE% [75]. When the drug concentration in the formulation is below its saturation solubility, no drug is expelled into the surrounding medium [76]. Therefore, the relatively low EE% obtained for FLC represents a limitation of the formulation and should be considered when evaluating its potential for drug delivery. These findings are also consistent with the exploratory molecular docking results.

The increase in FLC content in the formulation did not result in significant changes in EE% for either drug (*p* > 0.05). However, there was a substantial increase in DL% (*p* < 0.05), reaching twice the value observed in the formulation with the lowest FLC concentration. El-Hammadi et al. [77] also observed that DL% increases linearly with the amount of 5-fluorouracil, a hydrophilic drug, incorporated into the formulation. Considering that a high DL% is essential for drug delivery efficiency, especially in clinical studies where the amount and volume of the administered formulation are highly restricted, and that EE% remained unchanged, the NLC with the highest FLC concentration was selected as the best system [78].

The NLC-FR formulations showed no variation in appearance compared to NLCs. They did not cause significant changes (*p* > 0.05) in the hydrodynamic particle diameter, PDI, or zeta potential, indicating that the surface charges of the nanosystems are related to the total lipid composition (Table 3). This insignificant impact on the characteristics of NLC particles upon drug incorporation has also been observed in previous studies [60].

#### 3.5.4. FTIR

The FTIR technique was used to investigate possible molecular interactions between the components used to produce the nanocarriers. To determine the interaction of FLC and RIP with other materials, the spectra of NLCs containing the drugs (NLC-FR) were compared with those of NLCs without FLC and RIP. These results are illustrated in Figure 4.

The FTIR spectrum of bacuri butter (BB) in the infrared region showed an intense band at 1736 cm^−1^, due to the stretching vibration of the C=O double bond, which corresponds to triacylglycerols and carbonyls of lipid esters. C–H bending vibrations accounted for the band observed at 1466 cm^−1^ [79,80]. The spectrum also showed symmetric and asymmetric stretching bands of the—CH2 (2853 cm^−1^) and—CH3 (2920 cm^−1^) bonds, characteristic of saturated fatty acids. The band at 719 cm^−1^ is characteristic of angular deformation of the—(CH2) *n* > 3—group, indicating the presence of saturated chains, as well as the relationship between the acyl groups of saturated and cis-monounsaturated fatty acids [81]. These results confirm that BB is mainly composed of triacylglycerols and are consistent with those of other butters, such as cocoa butter, which has a similar fatty acid composition [22].

In the NLC and NLC-FR spectra, bands are observed that are broadly consistent with those in the BB spectrum, which is the main component of the nanoparticles. However, high-intensity bands (stretching—CH2 at 2853 cm^−1^, stretching—CH3 at 2920 cm^−1^, and C=O double bond stretching at 1736 cm^−1^) showed low intensities in the nanocarrier spectra, as did the broadening of the band at 1464 cm−1 and the absence of the band at 685 cm^−1^. The absence or broadening of bands in the NLC and NLC-FR spectra may indicate a less ordered structure or the amorphous state of the lipid matrix [82].

The spectra showed that the incorporation of FLC and RIP did not disturb the lipid structure of NLCs, confirming the compatibility of the formulation components, since the characteristic bands of NLCs (without drugs) were preserved [83]. The absence of specific drug peaks in the NLC-FR spectrum may be due to low levels of FLC and RIP in the formulation, or to drug encapsulation, likely achieved by dissolving the drugs in the lipid matrix [84,85]. However, a discrete peak is observed at 1622 cm^−1^ (1), likely due to the combined effect of the C=O stretching vibrations present in both drugs. In addition, there is a band at 1517 cm^−1^ (2), attributed to RIP, associated with the symmetric and asymmetric stretching vibrations of the aromatic ring, involving the methoxyl groups (—OCH3) attached to the benzene ring. According to Riberio et al. [86], these characteristics suggest possible electrostatic interactions between the drug and the free groups on NLCs, which could explain RIP’s greater affinity for NLCs. This hypothesis is further corroborated by the shift to a higher wavelength at 2920 cm^−1^ in NLC-FRs, compared to the BB spectrum at 2916 cm^−1^, which can be attributed to the encapsulation of RIP in nanoparticles and by the molecular docking study [87].

#### 3.5.5. DSC

The melting parameters of the drugs (FLC and RIP), the solid lipid used in NLCs (bacuri butter), drug-free lipid nanoparticles, and lipid nanoparticles containing FLC and RIP (NLC-FR) are shown in Figure 5. The butter showed a maximum endothermic peak at 52.07 °C, a value consistent with the melting point of tripalmitin, the main triacylglycerol present in this butter [88]. FLC showed two endothermic peaks, at 103.38 and 142.93 °C. The first peak is related to the loss of crystallization water, while the second corresponds to the melting point of the crystalline form of the drug [89]. RIP exhibited an intense, well-defined, and characteristic endothermic peak at 91.43 °C, attributed to the melting of the substance in its crystalline state.

Thermal analysis revealed that the melting point of NLCs is lower than that of isolated solid lipids, although these nanoparticles remain solid at body temperature [90]. This reduction can be attributed to the presence of the surfactant, which alters the crystalline structure of the lipid matrix, as well as to the increased surface-to-volume ratio in smaller particles, which favors a lower melting temperature [79]. DSC showed two distinct melting peaks at 45.41 °C and 51.11 °C for empty NLCs and at 47.03 °C and 50.37 °C for NLCs containing drugs (NLC-FR). These multiple peaks can be attributed to oil-rich domains dispersed in the lipid matrix, a typical result of the combination of solid and liquid lipids in nanostructured systems [91]. Additional endothermic peaks observed at 155.94 °C and 167.72 °C were attributed to mannitol, used as a cryoprotectant in the lyophilization process, in accordance with previous findings [92,93].

The enthalpy of fusion is directly proportional to the order of the crystal structure: the greater the disorder, the less the energy required for fusion. In this context, the reduced enthalpy values observed for NLCs (18.32 J/g) and NLC-FR (28.85 J/g), compared to isolated bacuri butter (52.07 J/g), indicate a lower crystallinity of the lipid matrix, likely due to structural defects. This behavior is characteristic of NLCs, in which the addition of liquid lipids disrupts the organization of the crystalline network, rendering it imperfect and favoring drug incorporation [94,95].

Considering the enthalpy of solid lipid as 100%, the calculated recrystallization index was 35.18% for empty NLCs and 55.40% for NLCs with drugs, indicating partial recrystallization of the lipid [96]. The increase in these values in the NLC-FR formulation suggests that the incorporation of FLC and RIP promoted structural reorganization, possibly by accelerating the polymorphic transition and favoring a more orderly rearrangement of the lipid phase. Furthermore, the data suggest that the drugs may have been partially adsorbed onto the nanoparticle surface, interacting with the surfactant layer and thereby modifying the molecular arrangement [97]. It is also noteworthy that there are no endothermic peaks corresponding to the drugs in the DSC curve of the NLC-FR formulation, suggesting that the active ingredients are amorphous and homogeneously dispersed in the lipid matrix, corroborating their efficient incorporation [82,98].

### 3.6. Physical Stability During Storage Time

Stability studies were performed using samples stored as aqueous suspensions under refrigerated conditions (5 ± 1 °C) due to the lipid nature of NLCs, which have a low melting point and cannot withstand exposure to high temperatures for prolonged periods [41]. During the 90 days of storage (Figure 6), NLC-FR remained physically stable, maintaining the characteristics of hydrodynamic particle diameter, PDI, and zeta potential. Although PDI changed significantly (*p* < 0.05), the values remained below 0.2, indicating high particle size homogeneity.

After 90 days, both drugs still had a DR% above 90% (94.7 ± 3.69% for FLC and 90.6 ± 4.99% for RIP), despite showing significant reductions (*p* < 0.05) compared to the initial values, indicating low degradation during the analytical period. In addition, only about 5.1% of FLC and 5.8% of RIP were released from NLC-FR after 90 days (Figure 6). These values are considered relatively low, especially considering that the NLCs were kept in colloidal aqueous suspension for an extended period. Drying the material could further increase its viability in an industrial context [51]. Therefore, considering all the results, the system demonstrates high stability under the storage conditions to which it was subjected.

### 3.7. In Vitro Release of FLC and RIP

An in vitro release assay was performed to evaluate drug release from NLC-FRs over time. According to Figure 7, the release of FLC and RIP was slower compared to the free drug solution (FLC-RIP), suggesting the potential of the tested formulations to sustain drug release. However, this difference is more pronounced with RIP. For FLC, a significant difference (*p* < 0.05) was observed only at the final collection point, at 10 h (53.63% for FLC-RIP solution and 48.28% for NLC-FR), which was expected, given that the EE% of the drug was less than 20%, indicating that a large part is free for diffusion. On the other hand, RIP showed a difference of more than 50% compared to the free drug solution at all points from 4 h onwards, reaching 15.78% at the end of the experiment, compared to 36.68% for FLC-RIP. This suggests a homogeneous distribution of the drug in the lipid matrix and its incorporation into the lipid core during cooling to form NLCs [68].

The difference in release rate between the drugs may also be related to their affinity for the lipid matrix. As fluconazole is a hydrophilic drug, it has low affinity for the lipids that make up the NLC matrix, resulting in greater diffusion into the aqueous receiving medium. In contrast, RIP, due to its greater solubility in the lipid matrix, remains longer, promoting a slower and more sustained release [41]. Previous studies have also identified a faster release of fluconazole from lipid nanoparticles, even with high EE% values [99,100]. These results corroborate the results of molecular docking studies.

To understand the mechanisms that control drug release, several kinetic models were applied to determine which model best represented the release profile of FLC and RIP. The best-fit kinetic model was determined based on the highest R2adj and MSC values and the lowest AIC value. As shown in Table S7 (Supplementary Material), the Korsmeyer–Peppas model provided the best fit for encapsulated FLC, while the Peppas–Sahlin 2 model was the most suitable for RIP in nanocarriers. In contrast, the first-order model was the best fit for the free drug solution (FLC-RIP). This difference in the kinetic models used for NLC-FR and FLC-RIP solutions suggests that the developed system was effective in modifying drug-release kinetics [101].

The first-order model, applied to free drug solutions, characterizes a release profile where the amount of drug released is directly proportional to the amount remaining in the dosage form. Consequently, the release rate decreases over time as the drug is released [102]. These systems initially release the drug rapidly, followed by slow release, resulting in a sharp drop to levels that may be insufficient to achieve therapeutic effects. As a consequence, these platforms require repeated administration and high doses to maintain concentrations within the desired therapeutic window, leading to low patient adherence to treatment [103].

As expected, FLC and RIP encapsulated in nanoparticles exhibited distinct release mechanisms due to their differences in solubility and affinity for the lipid matrix. The Peppas–Sahlin 2 model applied to RIP (NLC-FR) suggests that release was controlled by a combination of lipid matrix relaxation and drug diffusion [104]. According to Table S7 (Supplementary Material), the RIP release mechanism is predominantly driven by relaxation rather than diffusion (k2 > k1). This is corroborated by the fact that RIP is more concentrated in the core of the nanoparticles, suggesting that its release occurs mainly through relaxation of the lipid matrix, possibly induced by thermal stress during the assay, which can cause polymorphic changes and increase the flexibility of the matrix, preceding drug diffusion [101]. The values of the terms also indicate that, over time, the diffusion rate decreases, as suggested by the negative value of k1, while the rate of relaxation of the lipid matrix increases, as reflected in the positive value of k2 [105]. This indicates that, after the initial diffusion of the free fraction of RIP, the relaxation of the lipid matrix becomes the predominant mechanism, facilitating the continuous release of the drug through the concentration gradient.

The Korsmeyer–Peppas model was identified as the best kinetic fit for FLC encapsulated in NLC-FR. This model is widely used when the release mechanism is unknown or involves multiple drug-release processes [106]. The value *n*, as shown in Table S7 (Supplementary Material), was found to be greater than 0.43, suggesting non-Fickian release behavior. This indicates that drug release is regulated by multiple processes, with diffusion and relaxation of the lipid matrix playing key roles in controlling the release rate, both from the outermost layers of the lipid core of the nanoparticles and from the interfaces formed by the surfactant, to which the FLC is likely attached [107,108].

The good fit to the Peppas–Sahlin 2 model (R^2^adj = 0.984) provides a deeper understanding of the kinetics of FLC release in NLC-FR. The fact that k1 is greater than k2 indicates that the release of FLC from the nanocarrier matrix is predominantly controlled by the diffusion process, rather than the relaxation of the lipid matrix. This behavior differs from RIP release, in which matrix relaxation is the predominant process, corroborating the hypothesis that drugs are distributed differently within the lipid matrix.

### 3.8. Antifungal Activity

The MICs of FLC and RIP, administered individually, in combination, or co-encapsulated in NLC-FR, were determined in *Candida* spp. The FLC exhibited a significant antifungal effect, with MICs ranging from 4 to 64 μg/mL, while RIP showed no antifungal activity against the tested species. However, the combination of drugs increased FLC activity, especially against strains of *C. krusei*, a pathogen that is intrinsically resistant to FLC, suggesting that RIP may be useful as an adjuvant in the treatment of multidrug-resistant fungal infections [109].

According to Figure 8, the MICs of FLC, both alone and in combination with RIP (FLC–RIP), were significantly reduced in all strains evaluated when the drugs were encapsulated in the system (NLC-FR). At least an 8-fold reduction in MIC was observed for *C. albicans* ATCC 10231 strain, reaching reductions of up to 128-fold against the *C. krusei* ATCC 34135 strain. As expected, NLCs (without drugs) did not show antifungal activity, confirming that the improvement in fungicidal activity observed with the nanoparticles was exclusively due to the encapsulated drugs, not to the formulation components. These results suggest that the required therapeutic dose can be reduced, thereby minimizing the risk of adverse effects. One possible explanation for the enhanced antifungal activity observed for NLC-FR is the potential contribution of RIP to the modulation of fungal resistance mechanisms. The molecular docking analysis performed in this study indicated that RIP can interact with resistance-associated proteins, including Mdr1, Cdr1, and Cd2, sharing some interaction residues with previously reported efflux pump inhibitors. These computational findings provide a structural hypothesis for a possible interaction of RIP with efflux-related proteins and may help explain the potentiation of FLC activity observed experimentally. In addition, the hydrophobic surface of NLC-FR could facilitate FLC interaction with and penetration into fungal cells, potentially increasing intracellular drug accumulation [100,110]. However, the docking results do not demonstrate direct inhibition of efflux pump activity, nor do they establish that RIP increases intracellular FLC accumulation. Therefore, the enhanced antifungal activity observed for NLC-FR should be interpreted as being consistent with a possible modulation of efflux-mediated resistance rather than as definitive evidence of an efflux-inhibitory mechanism. Further experimental studies measuring efflux activity and intracellular FLC accumulation would be required to confirm this proposed mechanism. Other studies have reported increased antifungal activity of agents such as FLC following their encapsulation in NLCs [52,82,110].

### 3.9. Molecular Docking

Studies have shown that most clinically resistant isolates of *Candida* spp. overexpress genes encoding membrane transport proteins such as Cdr1, Cdr2, and Mdr1, which play a central role in resistance development because they are associated with reduced intracellular drug accumulation. The highly homologous *CdR1* and *CdR2* genes encode ATP-binding cassette (ABC) transporters, which use adenosine triphosphate (ATP) as an energy source for active transport. On the other hand, the MD1 gene encodes a Major Facilitator Superfamily (MFS) transporter that uses the transmembrane proton gradient as a driving force to expel the drug from the cell [9,111,112].

Several strategies have been explored for the development of new antifungal therapies, including the use of natural products, vaccines, and the combination of antifungals with non-antifungal substances capable of enhancing their efficacy [113]. Among these approaches, the use of resistance modulators such as haloperidol, dexamethasone, and verapamil, which act as efflux pump inhibitors in *Candida albicans*, stands out. Previous studies have shown that these compounds exhibited a synergistic effect with FLC, increasing the susceptibility of yeasts to treatment [48,114,115,116].

Based on these considerations, molecular docking was performed to evaluate the predicted binding modes and docking scores of the evaluated ligand to the target proteins [117]. The results were obtained using different scoring functions, in which lower values indicate more favorable predicted interactions between the ligand and the molecular target. A protein for which the compound showed consensus docking values comparable to or higher than those of the reference drug (FLC) was considered a potential molecular target for further analysis [31].

The results of molecular docking for the three target proteins, as well as the consensus docking values obtained through consensus calculations, are presented in Table S8 (Supplementary Material). It can be observed that RIP exhibited probabilities greater than 85% for all targets analyzed, exceeding those of the control drug (FLC), indicating higher predicted consensus docking values and a more favorable predicted interaction profile with the evaluated proteins. Among the other compounds tested, only verapamil did not show consensus docking values higher than those of FLC in the proteins Mdr1 and Cdr2, suggesting a less favorable predicted interaction profile for these targets. This result may be related to the distinct mechanism of action of verapamil, which, although widely used as an efflux pump inhibitor, acts primarily on P-glycoprotein, an ABC family transporter present in human cells. This protein differs structurally and functionally from transporters found in yeast, such as those represented by Mdr1 and Cdr1 [118,119,120]. Thus, the docking results highlight differences in the predicted interaction profiles of the evaluated compounds.

It is also noteworthy that RIP exhibited a high consensus docking value for the protein CdR2 gene, suggesting this protein may represent a candidate target for further investigation of RIP–protein interactions [121]. Thus, the molecular interactions established by RIP with targets related to the Mdr1, Cdr1, and Cdr2 efflux transporters were analyzed in detail and compared with those observed with the other drugs tested. The bonds and interactions formed are illustrated in Figures S9–S11 (Supplementary Material).

In this context, RIP interactions with the targets corresponding to the Mdr1, Cdr1, and CdR2 transporters are predominantly hydrogen bonds (represented by green dashed lines) and hydrophobic interactions (pink and lilac dashed lines). Most of these interactions occur between the carbon atoms (C) of the methoxyl group (OCH3) and the aromatic rings of the RIP chemical structure, thereby favoring the molecule’s fitting into the hydrophobic regions of the protein-binding cavity. Specific hydrogen bonds with key residues of the target proteins are also noteworthy. In MdR1, for example, a hydrogen bond involving the PHE389 residue was observed, as evidenced by the hydrogen atom (H) of the RIP carbonyl group (=O). In the proteins encoded by the CdR1 and CdR2 genes, hydrogen bonds were identified with residues ARG1405 and LYS52, respectively, involving carbon atoms in acyclic regions of the molecule, thereby reinforcing RIP’s versatility in establishing multiple favorable interactions. Together, these contribute to the predicted stabilization of RIP within the modeled interaction regions [122].

Although only a few interaction residues are shared between RIP and FLC, such as VAL79 in MdR1 and ILE39 and PHE50 in CdR2, these overlapping residues indicate similarities in the regions explored by the two compounds. The presence of hydrophobic and aromatic interactions within the binding cavities suggests that RIP may interact with regions involved in the recognition or transport of FLC. However, these docking results should be interpreted as a structural hypothesis rather than direct evidence of competitive inhibition or efflux-pump blockade. In particular, molecular docking alone cannot establish whether RIP inhibits transporter function, alters substrate transport, or increases intracellular FLC accumulation. Therefore, the present computational results provide complementary evidence supporting the possibility of interactions between RIP and resistance-associated proteins, while experimental efflux assays and cellular accumulation studies would be required to confirm the proposed mechanism. These findings are consistent with previous reports describing the potentiating effect of riparins in combination with FLC against *Candida* spp. [15,109]. These results are consistent with the findings of Costa et al. [15], who demonstrated the ability of RIP to enhance the activity of norfloxacin against a strain of S. aureus that overproduces the NorA efflux pump, also belonging to the MFS family [123]. RIP also enhanced the activity of norfloxacin against strains of Acinetobacter baumannii with efflux-mediated resistance, in which the presence of the adeABC gene was demonstrated, which encodes the AdeABC protein, a proton-motive force-dependent efflux pump belonging to the RND family [123]. The data obtained in this study support further investigation of RIP as a potential modulator of efflux-associated mechanisms, including those involving fungal transporters, but do not by themselves demonstrate efflux pump inhibition or reversal of FLC resistance.

Comparative analysis of interactive residues revealed that RIP shares key residues with drugs previously described as EIs, especially in proteins encoded by the MdR1 and CdR2 genes. In the MdR1 protein, RIP shared the VAL79 residue with verapamil and the ILE78 residue with dexamethasone, indicating partially overlapping predicted interaction profiles. In addition, it shared residues PHE238 and TRP239 with verapamil, which are involved in pi-alkyl interactions and hydrogen bonds, thereby indicating additional similarities in their predicted interaction profiles. In the CdR2 protein, RIP shared residues ILE39, PHE50, and CYS42 with haloperidol and residues ILE39, CYS42, VAL53, and PRO88 with dexamethasone, indicating overlapping predicted interactions within the modeled protein structure. On the other hand, in the CdR1 protein, no residues were shared between RIP and the other drugs evaluated, which may indicate a distinct binding conformation or an alternative mode of interaction. These results suggest partially overlapping predicted interaction profiles, especially with the MdR1 and CdR2 proteins, which are involved in efflux-based resistance.

## 4. Conclusions

In this study, NLCs containing fluconazole and Riparin B were developed and optimized using a systematic Quality-by-Design approach combined with Response Surface Methodology. This strategy enabled the identification and evaluation of formulation and process variables influencing the critical quality attributes of the NLCs and supported the development of an optimized formulation containing the selected active compounds.

The present study does not claim novelty for the antifungal activity of Riparin B, its potential involvement in efflux-mediated resistance, or the combination of Riparin B with fluconazole, which have been previously investigated. Instead, the contribution of the present work lies in the systematic integration of QbD/RSM-based formulation optimization, physicochemical characterization, drug encapsulation and release assessment, antifungal evaluation, and complementary molecular modeling within a single FLC–RIP NLC development strategy.

Molecular modeling provided an exploratory structural assessment of possible interactions of Riparin B with representative components of the lipid matrix and with fungal resistance-associated proteins. The docking results indicated favorable predicted interactions for Riparin B within the modeled lipid configuration and with the resistance-associated proteins evaluated; however, these findings should not be interpreted as direct thermodynamic evidence of drug–lipid affinity, quantitative predictors of encapsulation, or definitive evidence of efflux-pump inhibition.

Functionally, Riparin B alone did not exhibit relevant antifungal activity under the conditions evaluated, whereas its association with fluconazole potentiated the antifungal activity of the drug against the tested *Candida* spp. Incorporation of the combination into the NLC platform resulted in a further reduction in MIC values. These findings indicate that the optimized NLC platform may enhance the antifungal performance of the FLC–RIP combination and provide a formulation framework for further investigation.

## Figures and Tables

**Figure 1:**
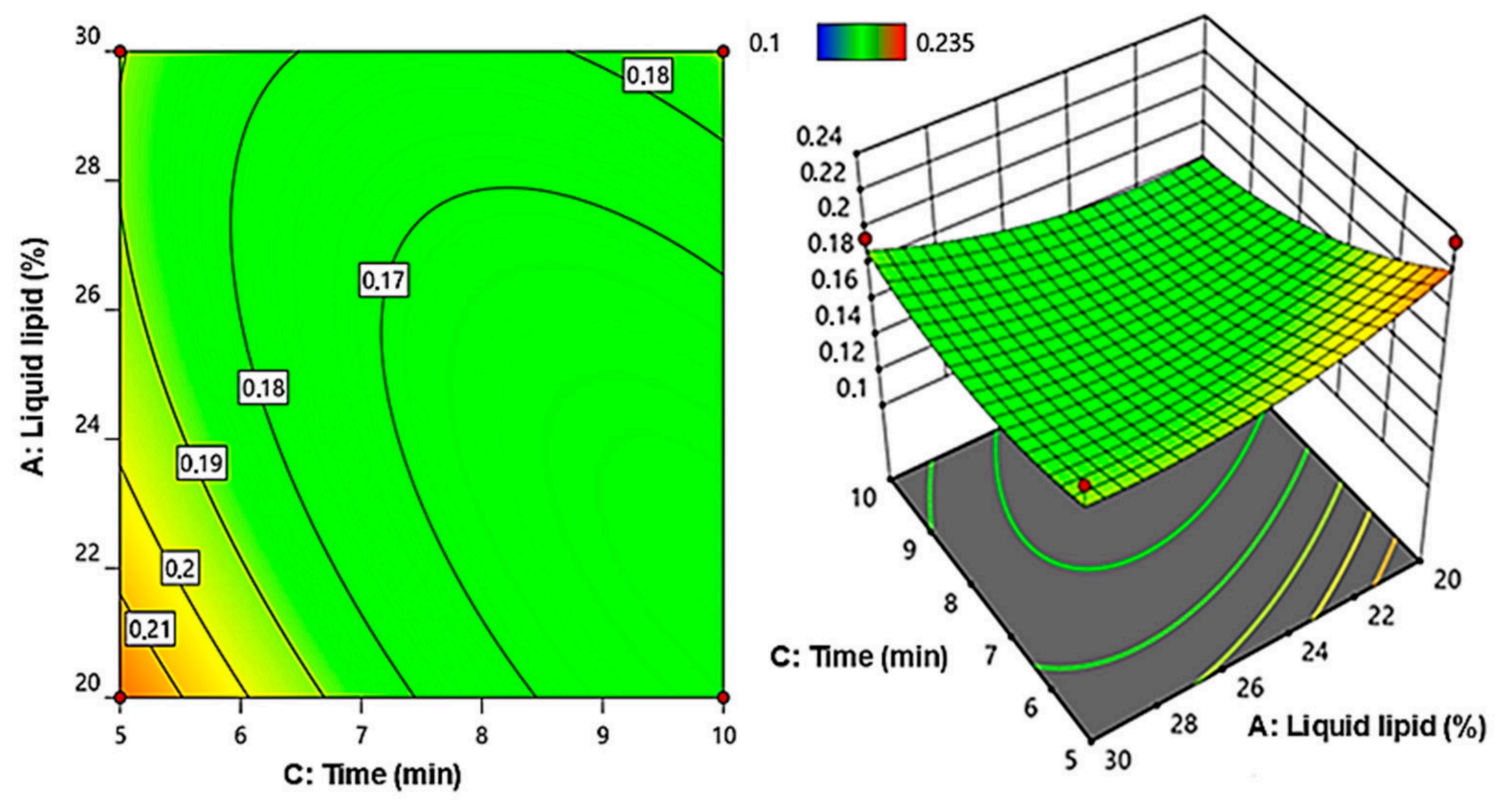
Contour and three-dimensional (3D) response surface graphs revealing the interaction of factors (AC) with the polydispersity index response (Y2).

**Figure 2:**
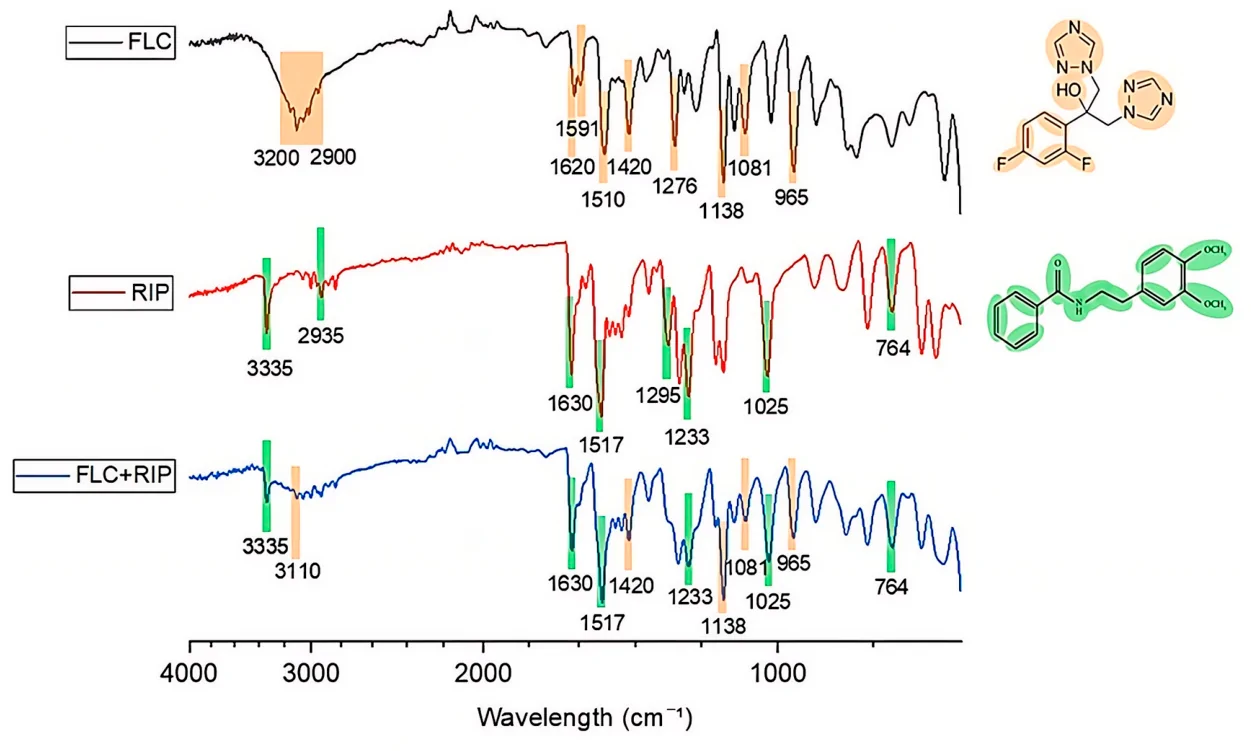
Infrared spectroscopy together with chemical structures of fluconazole (FLC) and riparin-B (RIP) alone and in a 1:1 *w*/*w* physical mixture (FLC + RIP).

**Figure 3:**
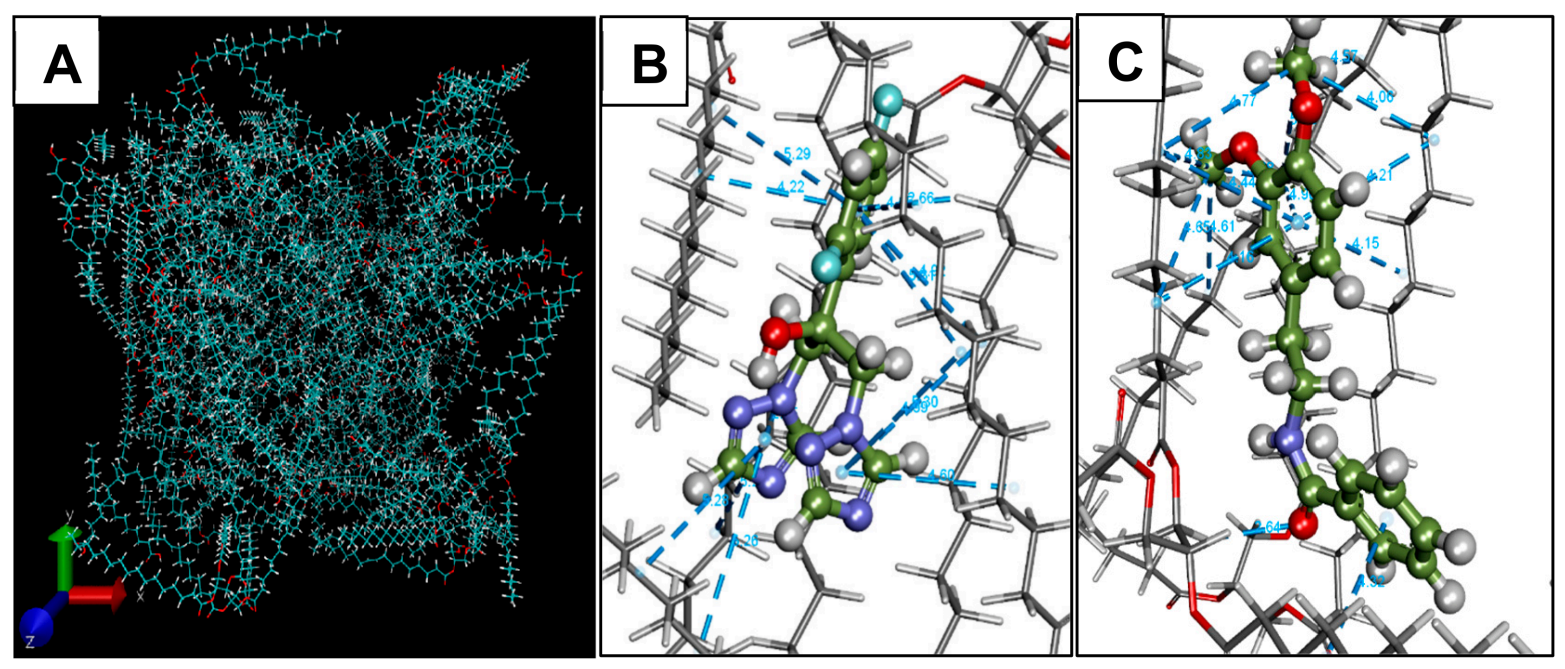
Three-dimensional representation of the lipid matrix (**A**) and docking conformations obtained for fluconazole (**B**) and riparin-B (**C**).

**Figure 4:**
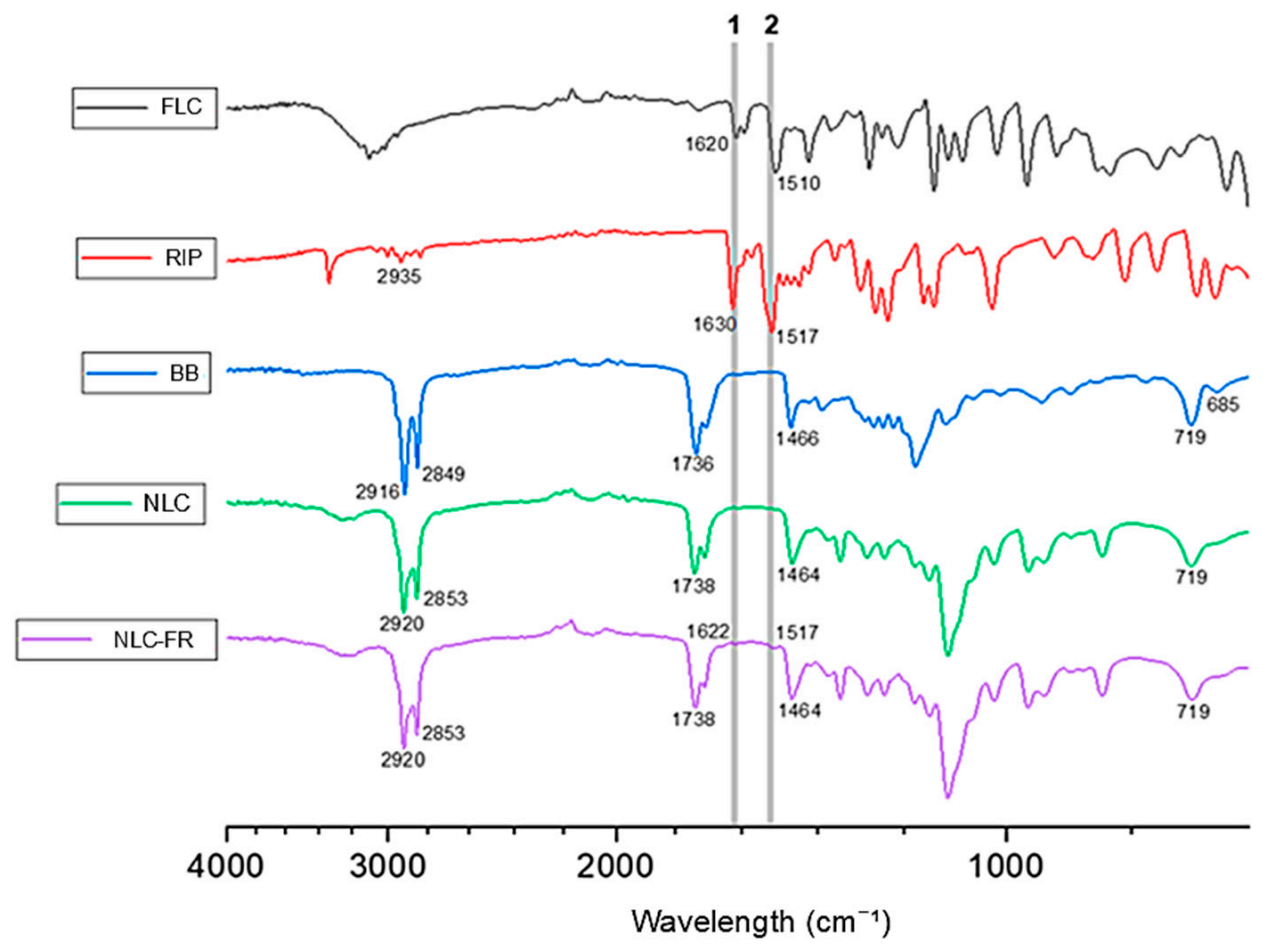
Infrared spectroscopy of fluconazole (FLC), riparin-B (RIP), bacuri butter (BB), blank NLCs, and NLCs loaded with drugs (NLC-FR).

**Figure 5:**
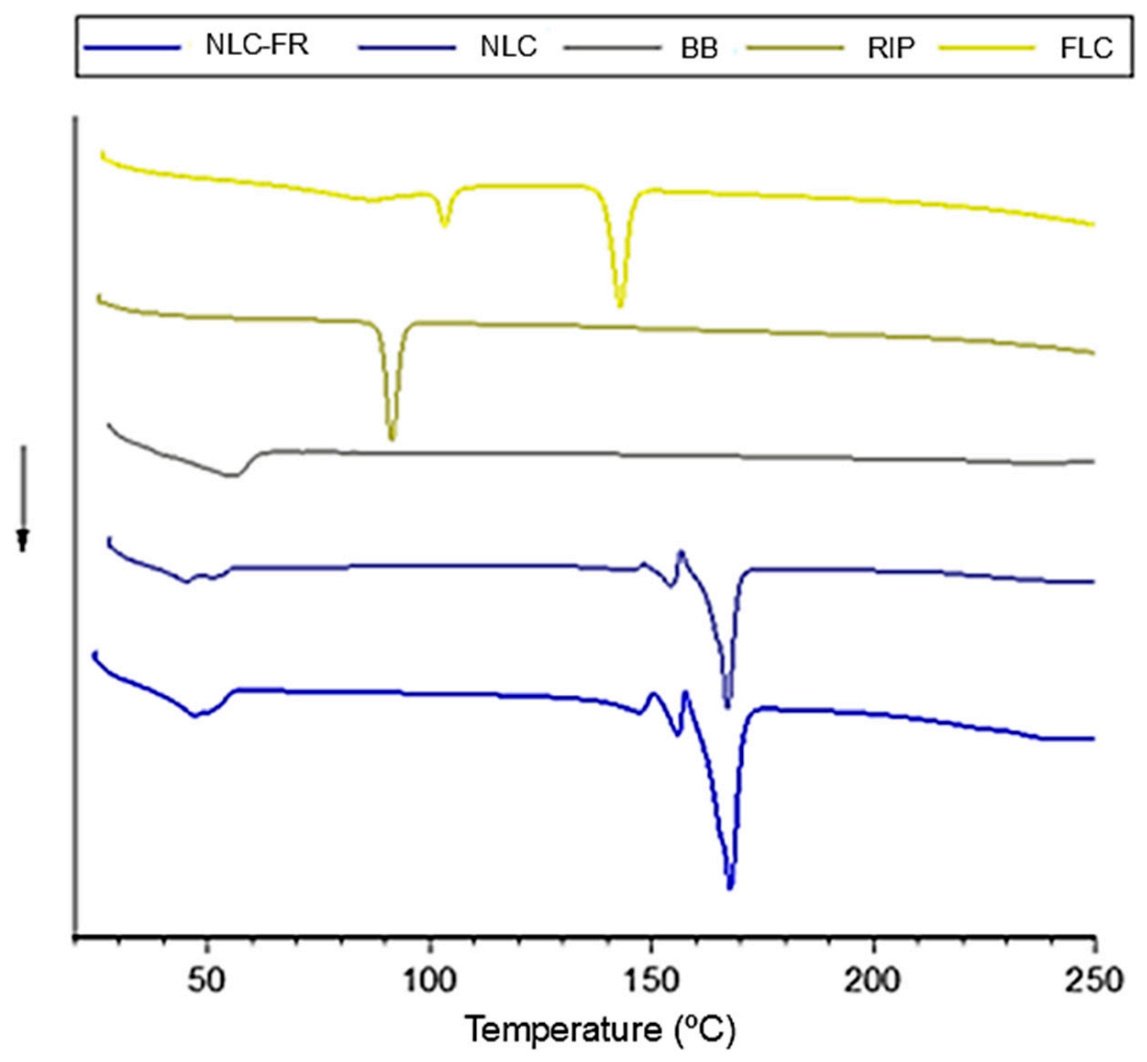
DSC of FLC, RIP, solid lipid (BB), and NLCs with and without drugs (NLC-FR).

**Figure 6:**
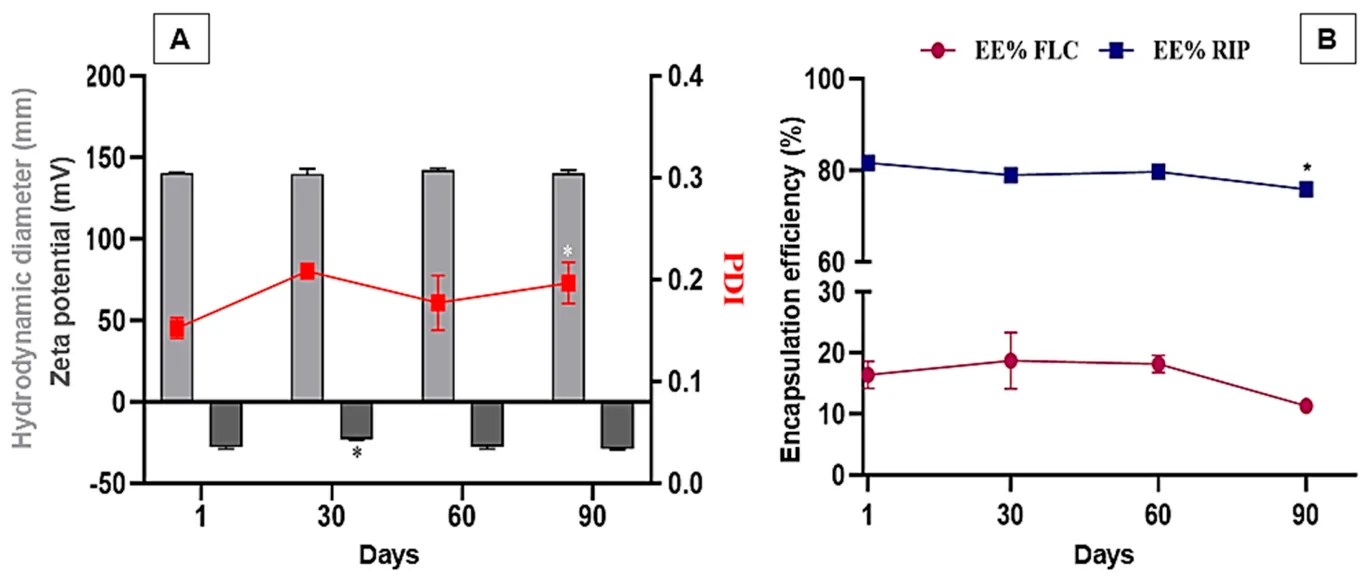
Hydrodynamic diameter, polydispersity index (PDI), zeta potential (**A**), and encapsulation efficiency (EE%) (**B**) of NLC-FR stored at 5.0 ± 2.0 °C for 90 days. Values represent the mean ± standard deviation of the mean; *n* = 3 for each formulation. Two-way ANOVA, followed by Tukey’s post-test. * *p* < 0.05.

**Figure 7:**
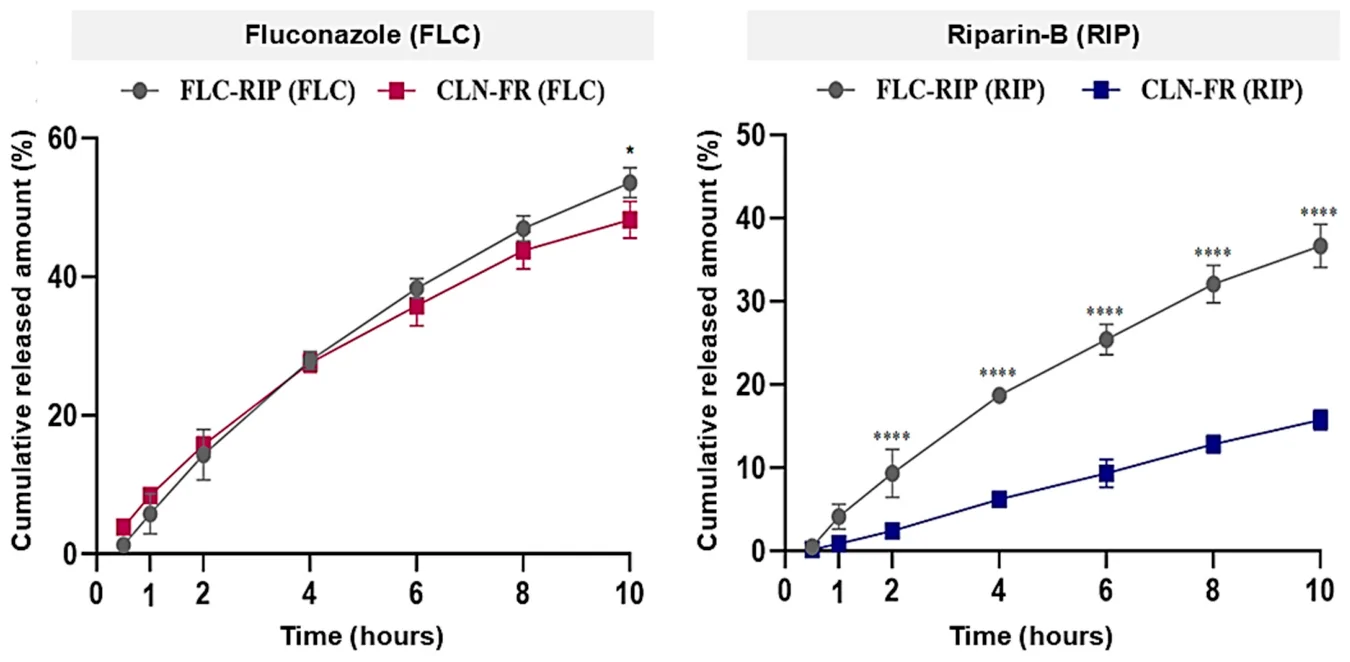
In vitro release profile of FLC and RIP from a solution of free drugs (FLC-RIP) and NLCs containing the drugs (NLC-FR) over 10 h. Values represent the mean ± standard deviation of the mean; *n* = 3 for each formulation. Two-way ANOVA, followed by Bonferroni post-test. * *p* < 0.05, **** *p* < 0.0001.

**Figure 8:**
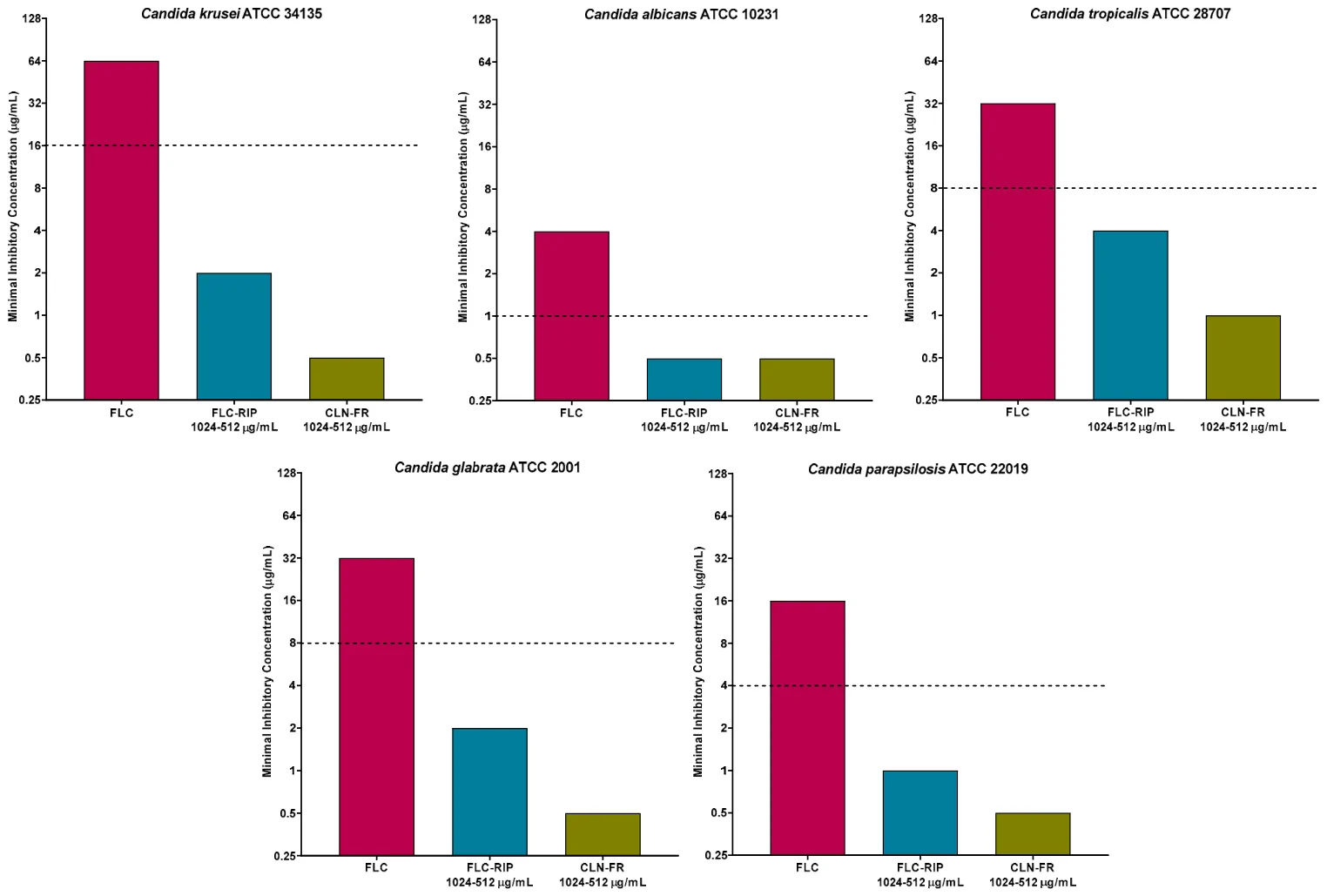
Effect of free Riparin B (RIP) and the NLC-FR nanosystem on the antifungal activity of fluconazole against the tested yeast strains. The dashed lines indicate the threshold value for consideration of an adjuvant effect, corresponding to a reduction by, at least, 4-fold of the antimicrobial MIC without RIP or CLN-FR (first column, in dark red).

**Table 1:** Independent and dependent factors (CQAs) and their respective levels for central composite design.

Central Composite Design (CCD)						
Code	Independent Factors Independent	Central Point	Factorial Points		Axial Points	
		0	−1	+1	−α	+α
A	Liquid lipid concentration ^a^	25	20	30	17	33
B	Surfactant concentration ^b^	4	3	5	2.3	5.7
C	Sonication time	7 min	5 min	10 min	3 min	12 min
**Critical Quality Attributes (CQAs)**						
Code	Dependent Factors			Objectives (CCD)		
Y1	Particle size (nm)			Minimize		
Y2	Polydispersity index			Minimize		
Y3	Zeta potential (mV)			At the limit		

a: Concentration relative to total lipid content. b: Concentration relative to aqueous phase.

**Table 2:** Results for size, polydispersity index (PDI), and zeta potential of the central composite design formulations.

Batch	Points	A	B	C	Size (nm)		PDI		Zeta Potential (mV)	
					Current Value	Predicted Value	Actual Value	Predicted Value	Actual Value	Predicted Value
1	Factorial	20	3	5	195.9 ± 3.60	204.42	0.160 ± 0.02	0.169	−26.6 ± 1.30	−26.72
2	Factorial	30	3	5	191.6 ± 2.32	189.51	0.139 ± 0.03	0.139	−26.3 ± 0.98	−25.77
3	Factorial	20	5	5	150.9 ± 2.61	156.36	0.235 ± 0.03	0.221	−24.9 ± 0.80	−25.05
4	Factorial	30	5	5	137.5 ± 2.37	141.46	0.202 ± 0.00	0.191	−26.9 ± 2.12	−26.65
5	Factorial	20	3	10	189.0 ± 3.52	191.51	0.124 ± 0.04	0.111	−27.5 ± 1.25	−26.68
6	Factorial	30	3	10	177.3 ± 0.69	176.61	0.144 ± 0.03	0.137	−26.3 ± 0.99	−25.72
7	Factorial	20	5	10	142.6 ± 2.60	143.45	0.156 ± 0.00	0.162	−24.7 ± 0.95	−25.01
8	Factorial	30	5	10	125.4 ± 2.50	128.55	0.195 ± 0.00	0.189	−26.2 ± 1.27	−26.61
9	Axial	17	4	7	175.3 ± 2.12	171.70	0.168 ± 0.01	0.171	−25.3 ± 0.49	−25.31
10	Axial	34	4	7	142.4 ± 2.38	146.36	0.166 ± 0.03	0.175	−24.9 ± 0.95	−25.36
11	Axial	25	2.32	7	213.6 ± 2.37	211.27	0.100 ± 0.02	0.099	−27.8 ± 1.11	−27.78
12	Axial	25	5.68	8	135.9 ± 1.81	130.53	0.178 ± 0.02	0.185	−28.6 ± 1.00	−27.12
13	Axial	25	4	3	186.5 ± 0.55	180.36	0.203 ± 0.02	0.208	−25.1 ± 0.40	−25.26
14	Axial	25	4	12	157.7 ± 1.87	157.13	0.147 ± 0.02	0.154	−24.9 ± 1.08	−25.18
15	Central	25	4	7	161.5 ± 1.25	159.77	0.145 ± 0.04	0.142	−27.9 ± 0.91	−27.45
16	Central	25	4	7	162.6 ± 0.26	159.77	0.126 ± 0.01	0.142	−28.6 ± 0.98	−27.45
17	Central	25	4	7	158.4 ± 2.41	159.77	0.132 ± 0.00	0.142	−25.8 ± 0.71	−27.45
18	Central	25	4	7	158.6 ± 1.63	159.77	0.156 ± 0.00	0.142	−27.1 ± 0.42	−27.45
19	Central	25	4	7	159.7 ± 0.40	159.77	0.127 ± 0.03	0.142	−24.4 ± 0.21	−27.45
20	Central	25	4	7	161.3 ± 3.69	159.77	0.159 ± 0.02	0.142	−26.0 ± 0.64	−26.72

A: Liquid lipid concentration (relative to total lipid amount). B: Surfactant concentration (relative to aqueous phase). C: Sonication time.

**Table 3:** Characterization data for NLC with (NLC-FR) and without fluconazole (FLC) and riparin-B (RIP), including hydrodynamic particle diameter, polydispersity index (PDI), zeta potential, encapsulation efficiency (EE%), and encapsulation capacity (DL%). NLC-FR (10/10 mg) corresponds to 10 mg of each drug; NLC-FR (20/10 mg) corresponds to 20 mg of FLC and 10 mg of RIP.

Sample	Hydrodynamic Diameter (nm)	PDI	Zeta Potential (mV)	EE (%)		DL (%)	
				FLC	RIP	FLC	RIP
NLC	143.1 ± 1.31	0.156 ± 0.01	−27.9 ± 0.97	-	-	-	-
NLC-FR (10/10 mg)	-	-	-	14.7 ± 4.61	78.9 ± 1.84	0.16 ± 0.06	0.70 ± 0.07
NLC-FR (20/10 mg)	140.7 ± 0.52	0.153 ± 0.01	−29.1 ± 2.26	16.4 ± 2.21	81.6 ± 0.65	0.32 ± 0.05	0.83 ± 0.04

## Data Availability

The raw data supporting the conclusions of this article will be made available by the authors upon request.

## Supplementary Materials

The following supporting information can be downloaded at: https://www.mdpi.com/article/10.3390/pharmaceutics18101229/s1. Table S1: Target Product Quality Profile (QTPP) for nanostructured lipid carriers based on natural lipids; Table S2: Critical Quality Attributes (CQAs) for nanostructured lipid carriers based on natural lipids; Figure S1: Ishikawa diagram showing the CMAs and CPPs that affect the development of nanostructured lipid carriers based on natural lipids; Figure S2: Initial risk assessment of nanostructured lipid carriers based on natural lipids based on the estimated interdependence between QTPPs and CQAs, and between CQAs and CMAs/CPPs; Table S3: Failure Mode and Effects Analysis (FMEA); Table S4: Analysis of variance of the regression model terms obtained from the central composite experimental design; Table S5: Regression equations of the analyzed responses (Y) as a function of the formulation variables (A, B, and C); Figure S3: Main effects for particle size (A) and zeta potential (B) as a function of the variables of the central composite experimental design; Table S6: Predicted and experimental values of optimized NLCs; Figure S4: Polarized light microscope images of fluconazole crystals (A), lipid mixture of bacuri butter and oleic acid containing 0.1% *w*/*w* (B), 0.2% *w*/*w* (C), and 0.3% *w*/*w* (D) fluconazole; Table S7: Evaluation of kinetic models applied to FLC and RIP release testing; Table S8: Predictive values and scores of drug interactions with resistance-related factors expressed by the MDR1, CDR1, and CDR2 genes; Figure S5: Molecular docking conformations with the lowest docking scores of fluconazole, Riparin B, haloperidol, dexamethasone, and verapamil with the protein expressed by the MDR1 gene; Figure S6: Molecular docking conformations with the lowest docking scores of fluconazole, Riparin B, haloperidol, dexamethasone, and verapamil with the protein expressed by the CDR1 gene; Figure S7: Molecular docking conformations with the lowest docking scores of fluconazole, Riparin B, haloperidol, dexamethasone, and verapamil with the protein expressed by the CDR2 gene.

## Abbreviations

The following abbreviations are used in this manuscript:

BB	Bacuri Butter
CCD	Central Composite Design
CMA	Critical Material Attribute
CPP	Critical Process Parameter
CQA	Critical Quality Attribute
DSC	Differential Scanning Calorimetry
EI	Efflux pump inhibitors
FLC	Fluconazole
FMEA	Failure Mode and Effects Analysis
FTIR	Fourier Transform Infrared Spectroscopy
NLC	Nanostructured Lipid Carrier
PDI	Polydispersity Index
QbD	Quality by Design
RIP	Riparin B
