# Supplementary material for: Co-Delivery of Fluconazole and Riparin B Using Nanostructured Lipid Carriers: A Quality-by-Design Approach for Enhanced Antifungal Activity
Source: Pharmaceutics. 2026 Sep 28;18(10):1229. doi: 10.3390/pharmaceutics18101229 (PMC13708304; doi:10.3390/pharmaceutics18101229)
Supplement: Supplementary file 1 [file pharmaceutics-18-01229-s001.zip › pharmaceutics-4552928-supplementary.pdf]

Table S1. Target Product Quality Profile (QTPP) for nanostructured lipid carriers based on natural lipids.

QTPP	Objective	Justification
Therapeutic indication	Persistent fungal infections not controlled by conventional FLC	Increased inherent and acquired resistance to FLC in *Candida* spp. species. Combination with natural or synthetic substances may increase treatment efficacy.
Route of administration	Local	Oral administration of FLC produces undesirable side effects, such as hepatotoxicity, gastrointestinal discomfort, and vomiting. The topical route offers ease of application, improved patient adherence, and enhanced therapeutic efficacy, achieving effects in a non-invasive manner.
Dosage form	Nanostructured lipid carriers based on natural lipids	Lipid-based systems increase drug solubility in the matrix and permeation through the skin layers, improving therapeutic efficacy. Natural lipids in NLCs confer intrinsic biological properties, such as skin hydration, as well as healing and anti-inflammatory effects.
Dosage	Maximum drug load in carriers	Reduction in the number of carriers needed to deliver a sufficient amount of drug with therapeutic action to the target site, as well as a decrease in drug waste during manufacturing.
Ideal pharmaceutical properties for the product intended for the market	1. Physical attributes	Uniform formulation, with pleasant odor and color that do not negatively affect organoleptic quality, aiming to increase patient adherence.
	2. Nanoparticles with a smaller and uniform size	The small size of NLCs ensures close contact between the lipid particles and the lipid bilayer of the stratum corneum, thereby facilitating permeation into the skin epithelium and deeper layers. In addition, the smaller particle size enables NLCs to be internalized into hyphal cells and into the fungal cell wall via endocytosis.
	3. Content: 90-110%	Content outside the limits affects the therapeutic efficacy of the formulation.
	4. Sustained release	Improve patient adherence by reducing the number of applications.

Stability	Stable under refrigerated conditions (4–8 °C)	Stability ensures an extended shelf life, consistent long-term efficacy, and retention of nanoparticles' intended properties. Changes in size, PDI, and zeta potential result in instability and heterogeneity of the system, manifesting before macroscopic changes are noticeable.

Table S2. Critical Quality Attributes (CQAs) for nanostructured lipid carriers based on natural lipids

CQAs	Objective	Justification
Particle size	NLCs smaller than 500 nm	After topical administration, the accumulation of FLC in the skin at adequate concentration levels is limited by the outermost layer of the skin, the stratum corneum. Particle sizes smaller than 500 nm are desirable for permeation into the deeper layers of the skin. In addition, the small size facilitates penetration of the fungal cell wall, causing it to rupture.
Polydispersity index (PDI)	PDI values less than 0.4	For a system with uniform particle size, the PDI should be close to 0.0. A PDI above 0.4 indicates a widely polydisperse system, with probable formation of aggregated particles.
Zeta potential	Above +30 mV or below -30 mV	NLCs with positive or negative net surface charges are physically stable, as this reduces the tendency for particle aggregation.
Encapsulated and/or solubilized FLC and RIP charge	Sufficient quantity to achieve maximum dosage without precipitation	The amount of encapsulated drug facilitates skin permeation and increases its apparent solubility due to supersaturation of the system.
Drug release profile	Sustained release compared to the free drug	Sustained drug release enables a prolonged therapeutic effect, reducing the frequency of application and increasing treatment adherence.

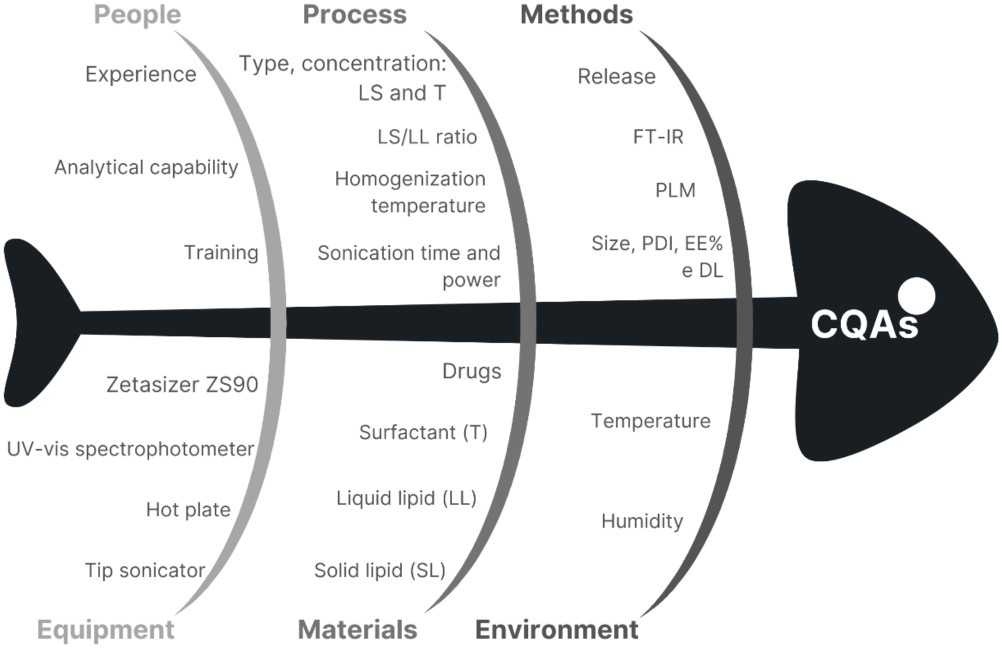

Figure S1. Ishikawa diagram showing the CMAs and CPPs that affect the development of nanostructured lipid carriers based on natural lipids.

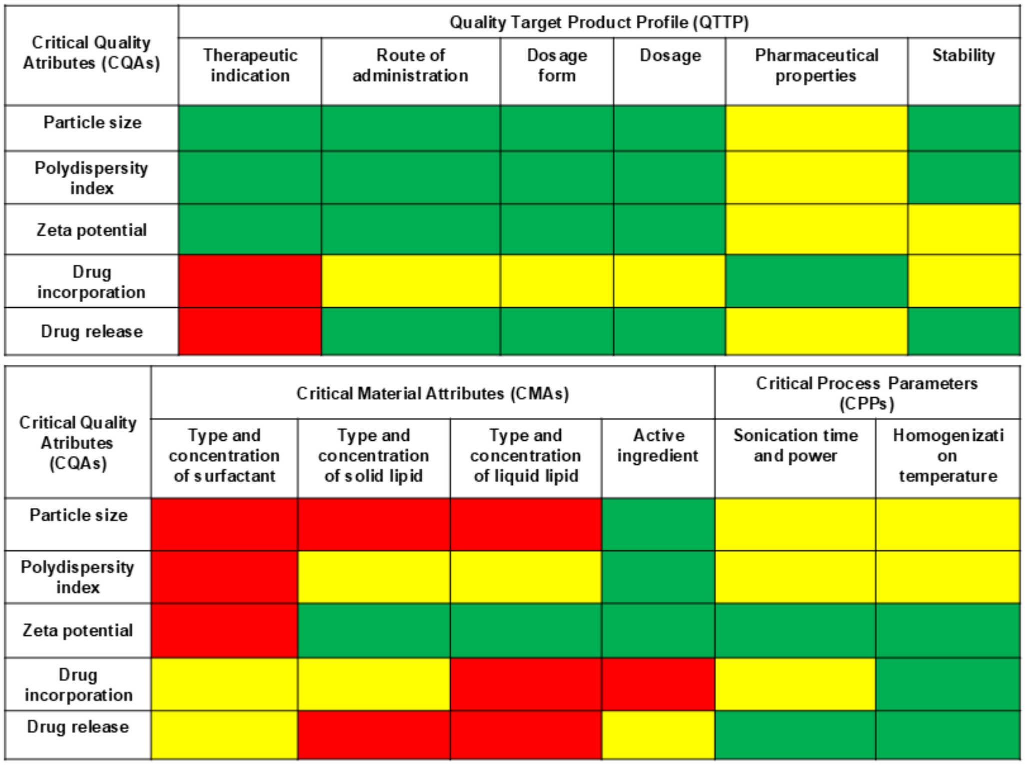

Figure S2. Initial risk assessment of nanostructured lipid carriers based on natural lipids based on the estimated interdependence between QTPPs and CQAs, and between CQAs and CMAs/CPPs

Table S3. Failure Mode and Effects Analysis (FMEA)

Dependent variables		Failure modes	Effects of failure	S	Potential cause of failure	O	Detectability method and control	D	RPN
**Characteristics of natural lipid-based nanoparticles**	**Particle size**	> 500 nm Non-reproducible batches	Ineffectiveness or limited benefit in antifungal action due to the absence of nanotechnological advantages	5	Insufficient stirring power<br>Insufficient agitation time	4	Dynamic light scattering (DLS)<br>Polarized light microscopy (PLM)<br>Standardization of methods	1	20
					High lipid concentration<br>Lipids with a very high melting point<br>Presence of unsolubilized drug crystals	5			25
			In topical/transdermal application, permeation through the skin may be limited	3	Insufficient agitation power<br>Insufficient agitation time	4	DLS<br>PLM<br><br>Standardization of production methods	1	12
					High lipid concentration<br>Lipids with a very high melting point<br>Presence of unsolubilized drug crystals	5			15
			Incorporation into semi-solid dosage forms may result in non-uniform doses.	3	Insufficient stirring power<br>Insufficient agitation time	4	DLS<br>PLM<br><br>Standardization of production methods	1	12
					High lipid concentration<br>Insufficient surfactant concentration<br>Presence of unsolubilized drug crystals	5			15
			Visible signs of instability, such as precipitation and coalescence, resulting from the presence of larger particles	3	Insufficient stirring power<br>Insufficient stirring time	4	DLS<br>Visualization<br><br>Standardization of production methods	1	12
					High lipid concentration<br>Insufficient surfactant concentration<br>Presence of unsolubilized drug crystals	5			15

Table S3. Failure mode and effect analysis (FMEA)

Dependent variables		Failure modes	Effects of failure	S	Potential cause of failure	O	Detectability method and control	D	RPN
Characteristics of natural lipid-based nanoparticles	**Polydispersity index**	Values >0.4	Ineffectiveness or limited benefit in antifungal action due to the inability to achieve uniform therapeutic doses	3	Insufficient agitation power<br>Insufficient agitation time	4	DLS<br><br>Standardization of production methods	1	12
					Inappropriate selection of surfactant<br>Inappropriate surfactant concentration<br>Presence of unsolved drug crystals	5			15
			Visible signs of instability, such as precipitation and coalescence	3	Insufficient stirring power<br>Insufficient stirring time	4	DLS<br><br>Standardization of production methods	1	12
					Inappropriate selection of surfactant<br>Inappropriate surfactant concentration<br>Presence of unsolved drug crystals	5			15
	**Zeta potential**	Above +30 mV or below -30 mV	Possible toxicity of nanoparticles caused by increased agglomeration (Berg et al., 2009)	5	Insufficient stirring power<br>Insufficient stirring time	3	Electrophoretic light scattering (ELS)<br><br>Standardization of production methods	1	15
					Inappropriate selection of surfactant<br>Inappropriate surfactant concentration<br>Inappropriate drug-lipid ratio	5			25
			Instability due to particle aggregation, leading to precipitation or coalescence	4	Insufficient stirring power<br>Insufficient stirring time	3	ELS<br><br>Standardization of production methods	2	9
					Inappropriate selection of surfactant<br>Inappropriate surfactant concentration<br>Inappropriate drug-lipid ratio	5			15

Table S3. Failure Mode and Effects Analysis (FMEA)

Dependent variables		Failure modes	Effects of failure	S	Potential cause of failure	O	Detectability method and control	D	RPN
**Characteristics of natural lipid-based nanoparticles**	**Encapsulated and solubilized FLC and RIP load**	Encapsulation efficiency <20% and presence of precipitated drug crystals	Ineffectiveness or limited benefit in antifungal action due to the absence of nanotechnological advantages	5	Inappropriate selection of lipid/surfactant<br>Inappropriate surfactant concentration<br>Inappropriate drug-lipid ratio<br>Low lipid concentration	3	Encapsulation efficiency assay (EE%)<br>Standardization of methods	3	60
			In topical/transdermal application, permeation through the skin may be limited	4	Inappropriate selection of lipid/surfactant<br>Inappropriate surfactant concentration<br>Inappropriate drug-lipid ratio<br>Low lipid concentration	3	EE%<br>Standardization of methods	3	36
			Incorporation into semi-solid pharmaceutical forms may result in non-uniform doses with the presence of precipitation	3	Inappropriate selection of lipid/surfactant<br>Inappropriate surfactant concentration<br>Inappropriate drug-lipid ratio<br>Low lipid concentration	3	Spectrophotometer<br>PLM<br>Standardization of methods	2	18
			Visible signs of instability due to drug precipitation	3	Inappropriate selection of lipid/surfactant<br>Inappropriate surfactant concentration<br>Inappropriate drug-lipid ratio<br>Low lipid concentration	3	EE%<br>Visualization<br>Standardization of methods	2	18
	**Drug release profile**	Absence or excess of the drug in the receiving compartment in a short period of time	Limited benefit due to very rapid release, not providing sustained benefits	5	Insufficient agitation power<br>Insufficient agitation time	3	Release test in Franz cell<br>Standardization of production methods	3	27
					Inappropriate selection of surfactant<br>Inappropriate surfactant concentration<br>Inappropriate drug-lipid ratio	4			60
			Limited benefit because drugs are not released in a way that achieves the desired therapeutic efficacy of the product	5	Insufficient agitation power<br>Insufficient agitation time	3	Franz cell release test<br>Standardization of production methods	3	36
					Inappropriate selection of surfactant<br>Inappropriate surfactant concentration<br>Inappropriate drug-lipid ratio	4			60

Table S3. Failure mode and effects analysis (FMEA)

Dependent variables		Failure modes	Effects of failure	S	Potential cause of failure	O	Detectability method and control	D	RPN
Materials	Surfactant	Inappropriate selection of surfactant	Inappropriate size<br>Heterogeneous size<br>Lack of stability<br>Changes in zeta potential<br>Toxicity	5	Lack of standardization<br>Inadequate HLB values<br>High production temperatures	3	DLS<br>ELS<br>Visualization	3	45
		Inadequate surfactant concentration	Inadequate or heterogeneous size<br>Lack of stability<br>Changes in zeta potential	5	Lack of standardization<br>Concentration below the critical micelle concentration	3	DLS<br>ELS<br>Visualization	5	75
	Lipid Solid	Inappropriate selection of solid lipid	Inappropriate or heterogeneous size<br>Drug release<br>Lack of stability<br>Low EE%<br>Drug expulsion	5	Lack of standardization<br>Solid lipid with a high melting point or molecular weight	3	DLS<br>Visualization<br>EE	2	30
		Inadequate concentration of solid lipid	Inadequate or heterogeneous size<br>Lack of stability<br>Low EE%<br>Low drug load	5	Lack of standardization<br>High or low concentrations	3	DLS<br>Visualization<br>EE%	5	45
	Liquid lipid	Inappropriate liquid lipid selection	Lack of stability<br>Unsolubilized drug crystals<br>Low EE%<br>Low drug load	5	Lack of standardization<br>Chemical structure with more or fewer unsaturations	3	DLS<br>Visualization<br>EE%	1	15
		Inappropriate solid/liquid lipid ratio	Inadequate or heterogeneous size<br>Lack of stability<br>Drug release	5	Lack of standardization<br>Solid/liquid lipid incompatibility<br>High lipid concentrations	3	DLS<br>Visualization<br>Franz cell release assay	5	75
	Active ingredient	No solubilization	Low EE%<br>Lack of stability<br>Limited antifungal action<br>Drug release	5	Lack of standardization<br>Inadequate concentration of formulation components	3	DLS/ELS<br>EE%<br>Antifungal activity	4	60

Table S3. Failure Mode and Effects Analysis (FMEA)

Dependent variables		Failure modes	Effects of failure	S	Potential cause of failure	O	Detectability method and control	D	RPN
Process	**Sonication**	High or low power<br>Short or long sonication time	Inappropriate or heterogeneous size<br>Lack of stability<br>Low drug loading	5	Lack of standardization<br>Failure of sonication equipment	3	DLS<br>EE%<br>Visualization	3	45
	**Temperature**	Low or high homogenization (emulsification) temperature	Inappropriate or heterogeneous size<br>Low drug load<br>Drug degradation	3	Error in temperature measurement<br>Overheating of sonication equipment	3	DLS<br>EE%	2	18

1-29 low risk	30–59 medium risk	60–125 high risk

Table S4. Analysis of variance of the regression model terms obtained from the central composite experimental design.

Terms	Y1			Y2			Y3		
	df	SQ	p	df	SQ	p	df	SQ	p
**Regression**	5	9589,53	0,000	6	0,018	0,000	6	17,97	0,037
**A**	1	766,04	0,000	1	0,000	0,803	1	0,358	0,542
**B**	1	7878,33	0,000	1	0,009	0,000	1	0,530	0,459
**C**	1	602,97	0,000	1	0,003	0,001	1	0,007	0,931
**AB**	-	-	-	-	-	-	1	3,25	0,083
**AC**	-	-	-	1	0,002	0,007	-		-
**A^2**	-	-	-	1	0,002	0,006	1	7,88	0,012
**B^2**	1	224,16	0,005	-	-	-	-		-
**C^2**	1	140,03	0,021	1	0,003	0,001	1	8,35	0,010
**Lack of adjustment**	9	250,73	0,080	8	0,001	0,774	8	5,00	0,859
**Pure error**	5	37,26		5	0,001		4	5,89	
**Total**	19	9877,52		19	0,019		18	28,86	

Y1: particle size, Y2: polydispersity index, Y3: zeta potential, A: liquid lipid concentration (relative to total lipid amount), B: surfactant concentration (relative to aqueous phase), C: sonication time; df: degrees of freedom; SQ: sum of squares; p: p-value

Table S5. Regression equations of the analyzed responses (Y) as a function of the formulation variables (A, B, and C).

Regression equation	R^2	R^2adj	R^2pred
$Y1 = 159,77 – 7,45\ A – 24,03\ B – 6,45\ C + 3,94\ B^2 + 2,77\ C^2$	0,971	0,960	0,913
$Y2 = 0,1422 – 0,0009\ A + 0,0258\ B – 0,0150\ C + 0,0141\ AC + 0,0105\ A^2 + 0,0120\ C^2$	0,899	0,852	0,760
$Y3 = 4,01 – 0,1620\ A + 0,1971\ B + 0,0221\ C + 0,7373\ A^2 + 0,6901\ C^2$	0,623	0,434	0,275

Y1: particle size, Y2: polydispersity index, Y3: zeta potential, A: liquid lipid concentration (relative to total lipid amount), B: surfactant concentration (relative to aqueous phase), C: Sonication time, R^2: Coefficient of determination, R^2adj: Adjusted coefficient of determination, R^2pred: Predicted coefficient of determination.

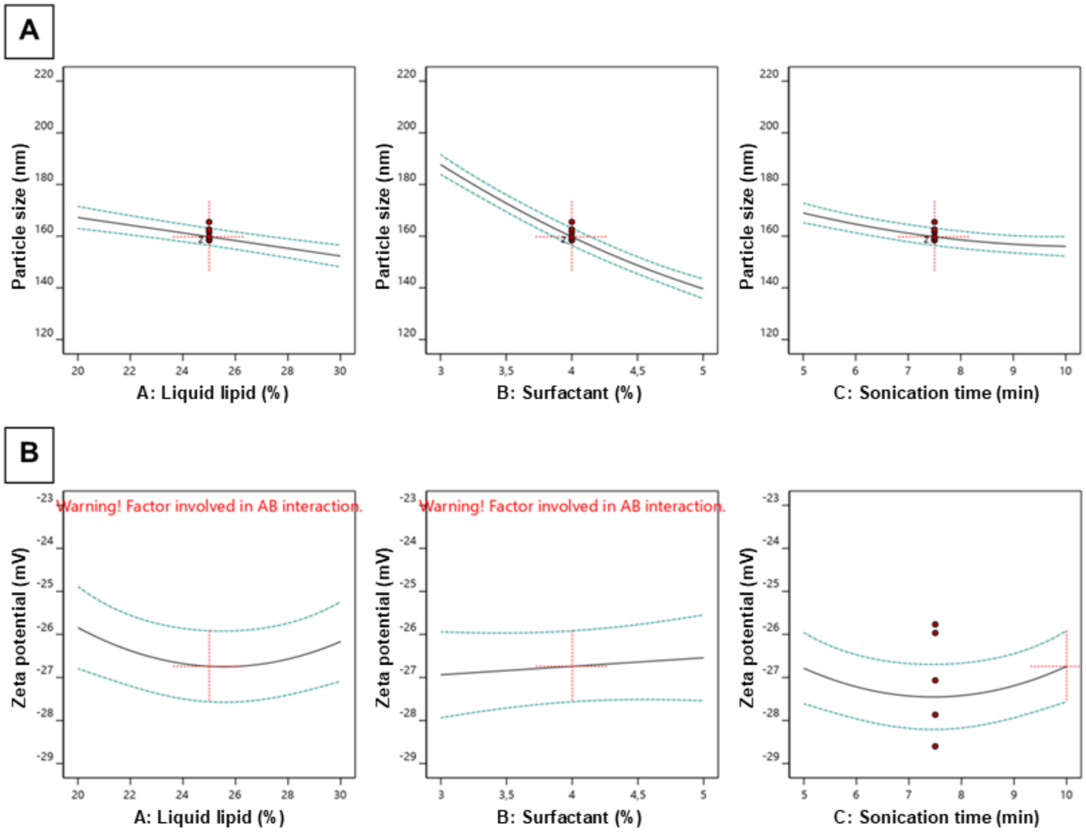

Figure S3. Main effects for particle size (A) and zeta potential (B) as a function of the variables of the central composite experimental design.

Table S6. Predicted and experimental values of optimized NLCs.

Responses	Ideal condition		
	Predicted values		Values obtained
	95% CI (lower)	95% CI (upper)	
Particle size (nm)	135,4	156,3	144,9 ± 2,25
PDI	0,122	0,180	0,153 ± 0,03
Zeta potential (mV)	-28,9	-24,4	-27,8 ± 1,81

CI: confidence index

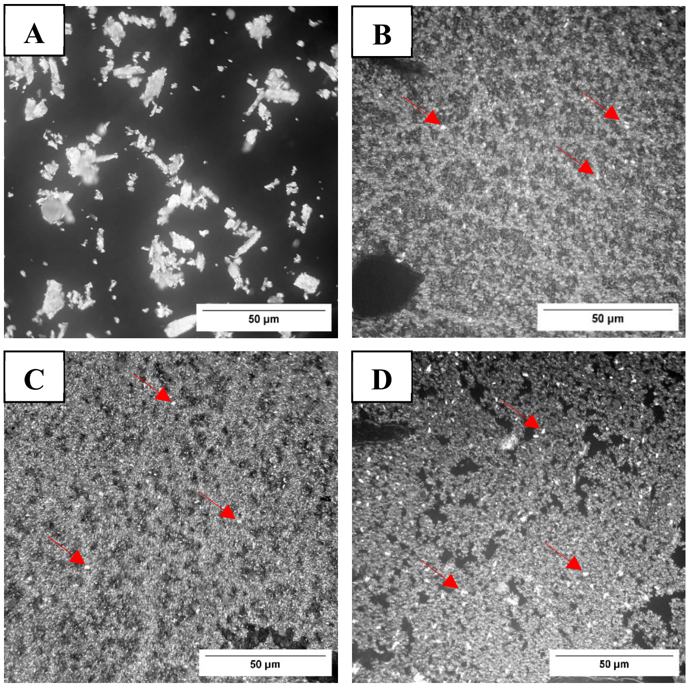

Figure S4. Polarized light microscope images of fluconazole crystals (A), lipid mixture of bacuri butter and oleic acid containing 0.1% w/w (B), 0.2% w/w (C), and 0.3% w/w (D) fluconazole.

Table S7. Evaluation of kinetic models applied to FLC and RIP release testing.

Model	Fluconazole (FLC)							
	FLC-RIP Solution				NLC-FR			
	R^2_{adj}	AIC	MSC	Parameters	R^2_{adj}	AIC	MSC	Parameters
**Zero order**	0,953	21,527	2,675	k0 = 5,802	0,843	25,293	1,459	k0 = 5,411
**First order**	0,992	12,401	4,500	k1 = 0,078	0,963	17,836	2,951	k1 = 0,071
**Higuchi**	0,871	26,623	1,656	kH = 15,615	0,944	20,166	2,485	kH = 14,732
**Korsmeyer-Peppas**	0,963	20,985	2,784	kKP = 6,951<br>n = 0,921	0,984	14,236	3,671	kKP = 9,983<br>n = 0,704
**Peppas-Sahlin 2**	0,976	18,908	3,199	k1 = 5,262<br>k2 = 3,893	0,984	14,333	3,651	k1 = 9,570<br>k2 = 1,941

Model	Riparin B (RIP)							
	FLC-RIP Solution				NLC-FR			
	R^2_{adj}	AIC	MSC	Parameters	R^2_{adj}	AIC	MSC	Parameters
**Zero order**	0,965	16,496	2,963	k0 = 3,931	0,989	2,615	4,191	k0 = 1,574
**First order**	0,989	9,328	4,396	k1 = 0,047	0,985	4,428	3,829	k1 = 0,017
**Higuchi**	0,854	23,670	1,528	kH = 10,553	0,769	18,233	1,068	kH = 4,167
**Korsmeyer-Peppas**	0,955	16,704	2,921	kKP = 4,819<br>n = 0,918	0,980	4,706	3,773	kKP = 1,211<br>n = 1,127
**Peppas-Sahlin 2**	0,974	14,795	3,303	k1 = 2,822<br>k2 = 2,907	0,991	1,222	4,470	k1 = -0,569<br>k2 = 1,781

R^2_{adj}: Adjusted coefficient of determination, AIC: Akaike Information Criterion, MSC: Model Selection Criterion, k0: Zero-order constant, k1: First-order constant, kH: Higuchi constant, kKP: Korsmeyer-Peppas constant, n: Release exponent, k1: Diffusion coefficient, k2: Relaxation coefficient.

Table S8. Predictive values and scores of drug interactions with resistance-related factors expressed by the MDR1, CDR1, and CDR2 genes.

**MDR1**									
**Compounds**	Autodock		Moldock		PLANTS		Rerank		**Penzyme**
	Valor	Probabilidade	Valor	Probabilidade	Valor	Probabilidade	Valor	Probabilidade	Probabilidade
Fluconazole	-3,24	0,580	-75,64	0,914	-61,68	0,827	-58,76	0,816	0,784
Riparin B	-4,04	0,723	**-82,79**	**1,000**	-60,87	0,816	**-71,97**	**1,000**	0,885
Haloperidol	-4,73	0,846	-71,21	0,860	**-74,56**	**1,000**	-64,04	0,890	**0,899**
Dexamethasone	**-5,59**	**1,000**	-57,69	0,697	-60,56	0,812	-59,37	0,825	0,833
Verapamil	-2,57	0,460	-69,91	0,844	-68,96	0,925	-57,82	0,803	0,758

**CDR1**									
**Compounds**	Autodock		Moldock		PLANTS		Rerank		**Penzyme**
	Valor	Probabilidade	Valor	Probabilidade	Valor	Probabilidade	Valor	Probabilidade	Probabilidade
Fluconazole	-3,38	0,677	-92,50	0,852	-65,11	0,875	-80,19	0,916	0,830
Riparin B	-4,53	0,908	-84,18	0,776	-63,07	0,848	-76,60	0,875	0,851
Haloperidol	-4,74	0,950	-84,38	0,777	**-74,41**	**1,000**	-76,92	0,878	**0,901**
Dexamethasone	**-4,99**	**1,000**	-73,42	0,676	-66,27	0,891	-74,35	0,849	0,854
Verapamil	-3,05	0,611	**-108,55**	**1,000**	-64,81	0,871	**-87,56**	**1,000**	0,871

**CDR2**									
**Compounds**	Autodock		Moldock		PLANTS		Rerank		**Penzyme**
	Valor	Probabilidade	Valor	Probabilidade	Valor	Probabilidade	Valor	Probabilidade	Probabilidade
Fluconazole	-3,39	0,630	-70,16	0,748	-64,67	0,889	-67,04	0,861	0,782
Riparin B	-5,18	0,963	**-93,85**	**1,000**	-67,77	0,932	**-77,90**	**1,000**	**0,974**
Haloperidol	-5,02	0,933	-70,99	0,756	**-72,73**	**1,000**	-64,50	0,828	0,879
Dexamethasone	**-5,38**	**1,000**	-69,77	0,743	-58,98	0,811	-70,20	0,901	0,864
Verapamil	-2,61	0,485	-85,27	0,909	-61,33	0,843	-60,03	0,771	0,752

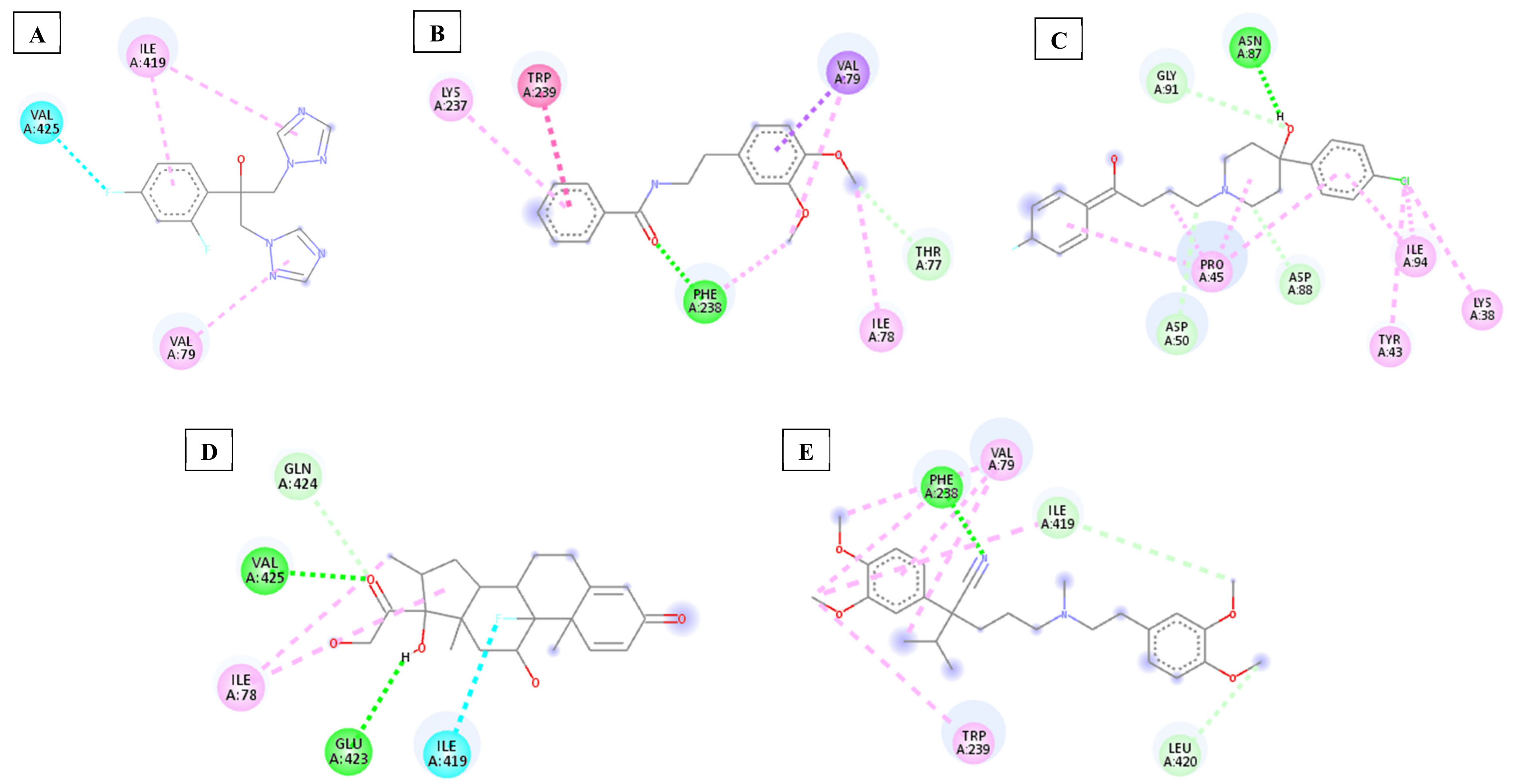

Figure S5. Molecular docking conformations with the lowest docking scores of fluconazole, Riparin B, haloperidol, dexamethasone, and verapamil with the protein expressed by the MDR1 gene

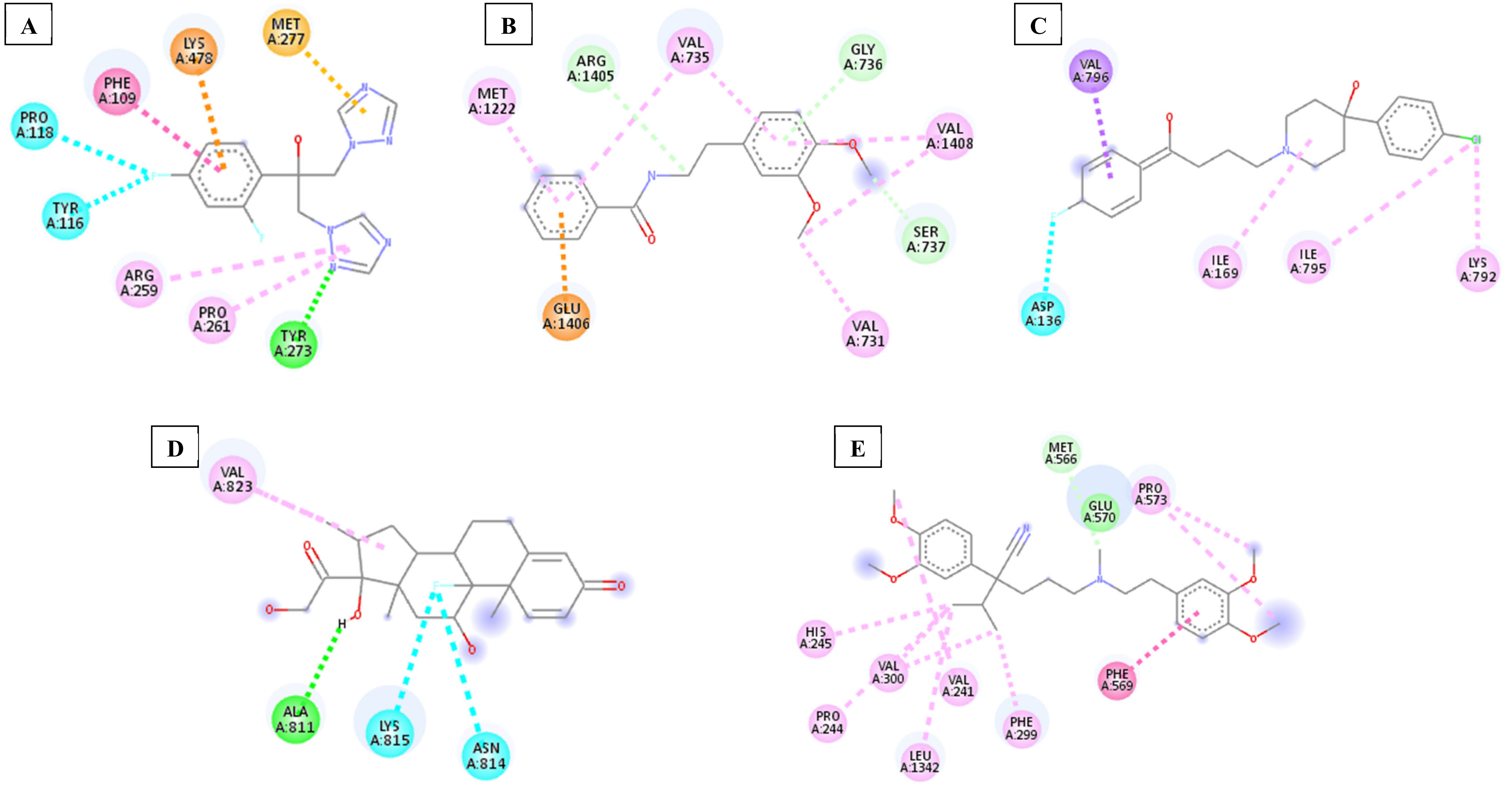

Figure S6. Molecular docking conformations with the lowest docking scores of fluconazole, Riparin B, haloperidol, dexamethasone, and verapamil with the protein expressed by the CDR1 gene

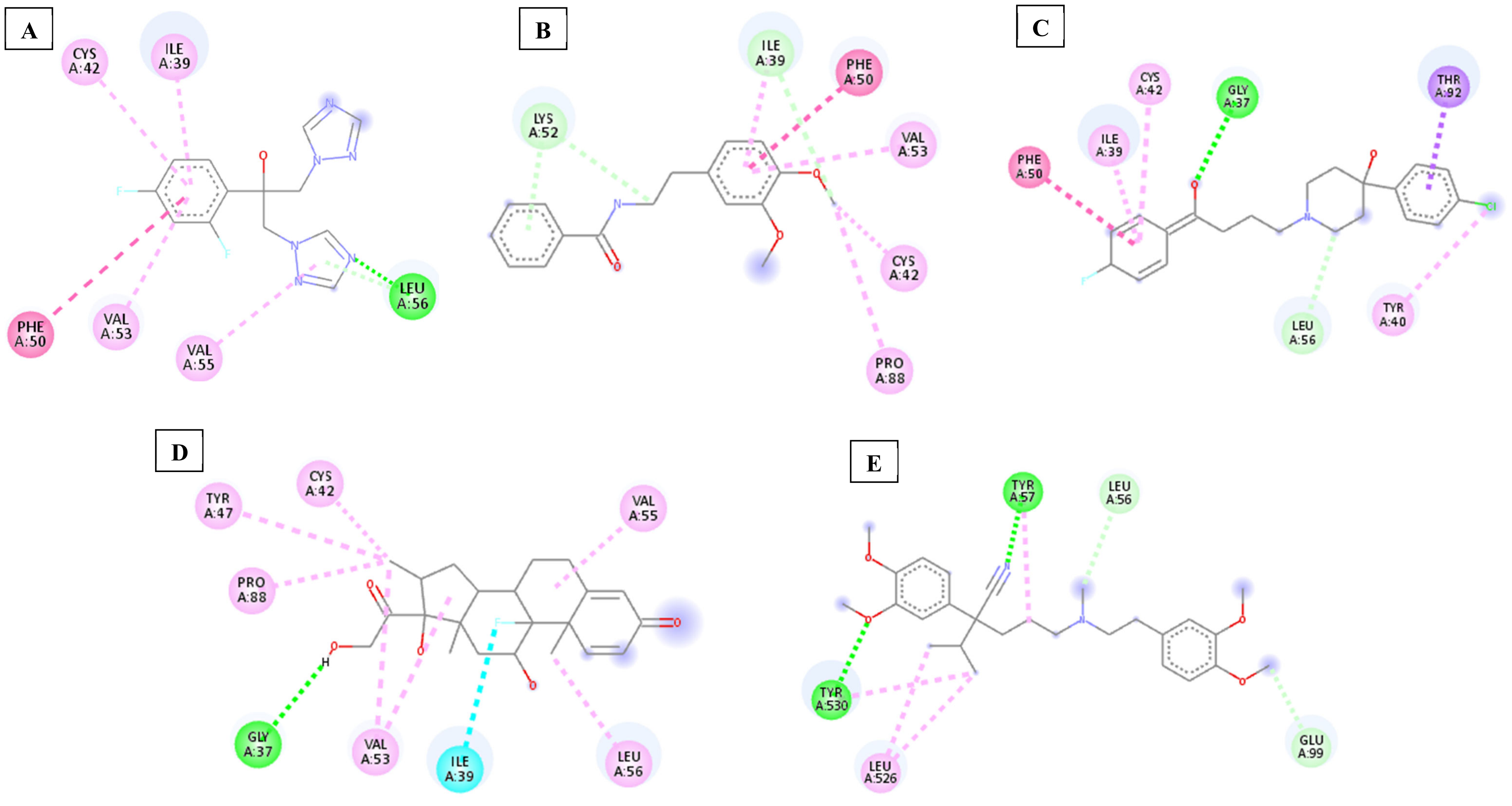

Figure S7. Molecular docking conformations with the lowest docking scores of fluconazole, Riparin B, haloperidol, dexamethasone, and verapamil with the protein expressed by the CDR2 gene.
